# Whole Genome Association Study of the Plasma Metabolome Identifies Metabolites Linked to Cardiometabolic Disease in Black Individuals

**DOI:** 10.1038/s41467-022-32275-3

**Published:** 2022-08-22

**Authors:** Usman A. Tahir, Daniel H. Katz, Julian Avila-Pachecho, Alexander G. Bick, Akhil Pampana, Jeremy M. Robbins, Zhi Yu, Zsu-Zsu Chen, Mark D. Benson, Daniel E. Cruz, Debby Ngo, Shuliang Deng, Xu Shi, Shuning Zheng, Aaron S. Eisman, Laurie Farrell, Michael E. Hall, Adolfo Correa, Russell P. Tracy, Peter Durda, Kent D. Taylor, Yongmei Liu, W. Craig Johnson, Xiuqing Guo, Jie Yao, Yii-Der Ida Chen, Ani W. Manichaikul, Frederick L. Ruberg, William S. Blaner, Deepti Jain, NHLBI Trans-Omics for Precision Medicine 1 Consortium, Namiko Abe, GonÃ§alo Abecasis, Francois Aguet, Christine Albert, Laura Almasy, Alvaro Alonso, Seth Ament, Peter Anderson, Pramod Anugu, Deborah Applebaum-Bowden, Kristin Ardlie, Dan Arking, Donna K. Arnett, Allison Ashley-Koch, Stella Aslibekyan, Tim Assimes, Paul Auer, Dimitrios Avramopoulos, Najib Ayas, Adithya Balasubramanian, John Barnard, Kathleen Barnes, R. Graham Barr, Emily Barron-Casella, Lucas Barwick, Terri Beaty, Gerald Beck, Diane Becker, Lewis Becker, Rebecca Beer, Amber Beitelshees, Emelia Benjamin, Takis Benos, Marcos Bezerra, Larry Bielak, Joshua Bis, Thomas Blackwell, John Blangero, Nathan Blue, Eric Boerwinkle, Donald W. Bowden, Russell Bowler, Jennifer Brody, Ulrich Broeckel, Jai Broome, Deborah Brown, Karen Bunting, Esteban Burchard, Carlos Bustamante, Erin Buth, Brian Cade, Jonathan Cardwell, Vincent Carey, Julie Carrier, April Carson, Cara Carty, Richard Casaburi, Juan P. Casas Romero, James Casella, Peter Castaldi, Mark Chaffin, Christy Chang, Yi-Cheng Chang, Daniel Chasman, Sameer Chavan, Bo-Juen Chen, Wei-Min Chen, Michael Cho, Seung Hoan Choi, Lee-Ming Chuang, Mina Chung, Ren-Hua Chung, Suzy Comhair, Matthew Conomos, Elaine Cornell, Carolyn Crandall, James Crapo, L. Adrienne Cupples, Joanne Curran, Jeffrey Curtis, Brian Custer, Coleen Damcott, Dawood Darbar, Sean David, Colleen Davis, Michelle Daya, Mariza de Andrade, Lisa de las Fuentes, Paul de Vries, Michael DeBaun, Ranjan Deka, Dawn DeMeo, Scott Devine, Huyen Dinh, Harsha Doddapaneni, Qing Duan, Shannon Dugan-Perez, Ravi Duggirala, Susan K. Dutcher, Charles Eaton, Lynette Ekunwe, Adel El Boueiz, Patrick Ellinor, Leslie Emery, Serpil Erzurum, Charles Farber, Jesse Farek, Tasha Fingerlin, Matthew Flickinger, Myriam Fornage, Nora Franceschini, Chris Frazar, Mao Fu, Stephanie M. Fullerton, Lucinda Fulton, Stacey Gabriel, Weiniu Gan, Shanshan Gao, Yan Gao, Margery Gass, Heather Geiger, Bruce Gelb, Mark Geraci, Soren Germer, Auyon Ghosh, Richard Gibbs, Chris Gignoux, Mark Gladwin, David Glahn, Stephanie Gogarten, Da-Wei Gong, Harald Goring, Sharon Graw, Kathryn J. Gray, Daniel Grine, Colin Gross, C. Charles Gu, Yue Guan, Namrata Gupta, Jeff Haessler, Yi Han, Patrick Hanly, Daniel Harris, Nicola L. Hawley, Jiang He, Ben Heavner, Susan Heckbert, Ryan Hernandez, David Herrington, Craig Hersh, Bertha Hidalgo, James Hixson, Brian Hobbs, John Hokanson, Elliott Hong, Karin Hoth, Chao Hsiung, Jianhong Hu, Yi-Jen Hung, Haley Huston, Chii Min Hwu, Marguerite Ryan Irvin, Rebecca Jackson, Cashell Jaquish, Jill Johnsen, Andrew Johnson, Rich Johnston, Kimberly Jones, Hyun Min Kang, Robert Kaplan, Sharon Kardia, Shannon Kelly, Eimear Kenny, Michael Kessler, Alyna Khan, Ziad Khan, Wonji Kim, John Kimoff, Greg Kinney, Barbara Konkle, Charles Kooperberg, Holly Kramer, Christoph Lange, Ethan Lange, Leslie Lange, Cathy Laurie, Cecelia Laurie, Meryl LeBoff, Jiwon Lee, Sandra Lee, Wen-Jane Lee, Jonathon LeFaive, David Levine, Dan Levy, Joshua Lewis, Xiaohui Li, Yun Li, Henry Lin, Honghuang Lin, Xihong Lin, Simin Liu, Yu Liu, Ruth J. F. Loos, Steven Lubitz, Kathryn Lunetta, James Luo, Ulysses Magalang, Michael Mahaney, Barry Make, Alisa Manning, JoAnn Manson, Lisa Martin, Melissa Marton, Susan Mathai, Rasika Mathias, Susanne May, Patrick McArdle, Merry-Lynn McDonald, Sean McFarland, Stephen McGarvey, Daniel McGoldrick, Caitlin McHugh, Becky McNeil, Hao Mei, James Meigs, Vipin Menon, Luisa Mestroni, Ginger Metcalf, Deborah A. Meyers, Emmanuel Mignot, Julie Mikulla, Nancy Min, Mollie Minear, Ryan L. Minster, Braxton D. Mitchell, Matt Moll, Zeineen Momin, May E. Montasser, Courtney Montgomery, Donna Muzny, Josyf C. Mychaleckyj, Girish Nadkarni, Rakhi Naik, Take Naseri, Sergei Nekhai, Sarah C. Nelson, Bonnie Neltner, Caitlin Nessner, Deborah Nickerson, Osuji Nkechinyere, Kari North, Jeff O’Connell, Tim O’Connor, Heather Ochs-Balcom, Geoffrey Okwuonu, Allan Pack, David T. Paik, Nicholette Palmer, James Pankow, George Papanicolaou, Cora Parker, Gina Peloso, Juan Manuel Peralta, Marco Perez, James Perry, Ulrike Peters, Patricia Peyser, Lawrence S. Phillips, Jacob Pleiness, Toni Pollin, Wendy Post, Julia Powers Becker, Meher Preethi Boorgula, Michael Preuss, Bruce Psaty, Pankaj Qasba, Dandi Qiao, Zhaohui Qin, Nicholas Rafaels, Laura Raffield, Mahitha Rajendran, Vasan S. Ramachandran, D. C. Rao, Laura Rasmussen-Torvik, Aakrosh Ratan, Susan Redline, Robert Reed, Catherine Reeves, Elizabeth Regan, Alex Reiner, Muagututiâ€ã Sefuiva Reupena, Ken Rice, Rebecca Robillard, Nicolas Robine, Dan Roden, Carolina Roselli, Ingo Ruczinski, Alexi Runnels, Pamela Russell, Sarah Ruuska, Ester Cerdeira Sabino, Danish Saleheen, Shabnam Salimi, Sejal Salvi, Steven Salzberg, Kevin Sandow, Vijay G. Sankaran, Jireh Santibanez, Karen Schwander, David Schwartz, Frank Sciurba, Christine Seidman, Jonathan Seidman, FrÃ©dÃ©ric SÃ©riÃ¨s, Vivien Sheehan, Stephanie L. Sherman, Amol Shetty, Aniket Shetty, Wayne Hui-Heng Sheu, M. Benjamin Shoemaker, Brian Silver, Edwin Silverman, Robert Skomro, Albert Vernon Smith, Jennifer Smith, Josh Smith, Nicholas Smith, Tanja Smith, Sylvia Smoller, Beverly Snively, Michael Snyder, Tamar Sofer, Nona Sotoodehnia, Adrienne M. Stilp, Garrett Storm, Elizabeth Streeten, Jessica Lasky Su, Yun Ju Sung, Jody Sylvia, Adam Szpiro, Daniel Taliun, Hua Tang, Margaret Taub, Matthew Taylor, Simeon Taylor, Marilyn Telen, Timothy A. Thornton, Machiko Threlkeld, Lesley Tinker, David Tirschwell, Sarah Tishkoff, Hemant Tiwari, Catherine Tong, Michael Tsai, Dhananjay Vaidya, David Van Den Berg, Peter VandeHaar, Scott Vrieze, Tarik Walker, Robert Wallace, Avram Walts, Fei Fei Wang, Heming Wang, Jiongming Wang, Karol Watson, Jennifer Watt, Daniel E. Weeks, Joshua Weinstock, Bruce Weir, Scott T. Weiss, Lu-Chen Weng, Jennifer Wessel, Cristen Willer, Kayleen Williams, L. Keoki Williams, Carla Wilson, Lara Winterkorn, Quenna Wong, Joseph Wu, Huichun Xu, Lisa Yanek, Ivana Yang, Ketian Yu, Seyedeh Maryam Zekavat, Yingze Zhang, Snow Xueyan Zhao, Wei Zhao, Xiaofeng Zhu, Elad Ziv, Michael Zody, Sebastian Zoellner, Claude Bouchard, Mark A. Sarzynski, Stephen S. Rich, Jerome I. Rotter, Thomas J. Wang, James G. Wilson, Clary B. Clish, Pradeep Natarajan, Robert E. Gerszten

**Affiliations:** 1 Division of Cardiovascular Medicine, Beth Israel Deaconess Medical Center, Harvard Medical School, Boston, MA US; 2 Broad Institute of Harvard and MIT, Cambridge, MA US; 3 University of Mississippi Medical Center, Jackson, MS US; 4 Department of Pathology Laboratory Medicine, Larner College of Medicine, University of Vermont, Burlington, VT US; 5 The Institute for Translational Genomics and Population Sciences, Department of Pediatrics, The Lundquist Institute for Biomedical Innovation at Harbor UCLA Medical Center, Torrance, CA US; 6 Department of Medicine, Division of Cardiology, Duke Molecular Physiology Institute, Duke University Medical Center, Durham, NC US; 7 Department of Biostatistics, University of Washington, Seattle, WA US; 8 Center for Public Health Genomics, University of Virginia, Charlottesville, Virginia US; 9 Division of Biostatistics and Epidemiology, Department of Public Health Sciences, University of Virginia, Charlottesville, Virginia US; 10 Section of Cardiovascular Medicine, Boston University School of Medicine and Boston Medical Center, Boston, MA US; 11 Columbia University Medical Center, New York, NY US; 12 University of Washington, Seattle, Washington US; 13 Human Genomic Laboratory, Pennington Biomedical Research Center, Baton Rouge, LA US; 14 Department of Exercise Science, University of South Carolina, Columbia, SC US; 15 Department of Medicine, UT Southwestern Medical Center, Dallas, TX US; 16 Cardiovascular Research Center, Massachusetts General Hospital, Harvard Medical School, Boston, MA US; 17 New York Genome Center, New York, New York 10013 US; 18 University of Michigan, Ann Arbor, Michigan 48109 US; 19 Broad Institute, Cambridge, Massachusetts 2142 US; 20 Cedars Sinai, Boston, Massachusetts 2114 US; 21 Children’s Hospital of Philadelphia, University of Pennsylvania, Philadelphia, Pennsylvania 19104 US; 22 Emory University, Atlanta, Georgia 30322 US; 23 University of Maryland, Baltimore, Maryland 21201 US; 24 University of Mississippi, Jackson, Mississippi 38677 US; 25 National Institutes of Health, Bethesda, Maryland 20892 US; 26 Johns Hopkins University, Baltimore, Maryland 21218 US; 27 University of Kentucky, Lexington, Kentucky 40506 US; 28 Duke University, Durham, North Carolina 27708 US; 29 University of Alabama, Birmingham, Alabama 35487 US; 30 Stanford University, Stanford, California 94305 US; 31 Medical College of Wisconsin, Milwaukee, Wisconsin 53211 US; 32 Providence Health Care, Medicine, Vancouver, CA US; 33 Baylor College of Medicine Human Genome Sequencing Center, Houston, Texas 77030 US; 34 Cleveland Clinic, Cleveland, Ohio 44195 US; 35 Tempus, University of Colorado Anschutz Medical Campus, Aurora, Colorado 80045 US; 36 Columbia University, New York, New York 10032 US; 37 The Emmes Corporation, LTRC, Rockville, Maryland 20850 US; 38 Cleveland Clinic, Quantitative Health Sciences, Cleveland, Ohio 44195 US; 39 Johns Hopkins University, Medicine, Baltimore, Maryland 21218 US; 40 National Heart, Lung, and Blood Institute, National Institutes of Health, Bethesda, Maryland 20892 US; 41 Boston University, Massachusetts General Hospital, Boston University School of Medicine, Boston, Massachusetts 2114 US; 42 University of Pittsburgh, Pittsburgh, Pennsylvania 15260 US; 43 FundaÃ§Ã£o de Hematologia e Hemoterapia de Pernambuco - Hemope, Recife, 52011-000 BR Brazil; 44 University of Washington, Cardiovascular Health Research Unit, Department of Medicine, Seattle, Washington 98195 US; 45 University of Texas Rio Grande Valley School of Medicine, Human Genetics, Brownsville, Texas 78520 US; 46 University of Utah, Obstetrics and Gynecology, Salt Lake City, Utah 84132 US; 47 University of Texas Health at Houston, Houston, Texas 77225 US; 48 Wake Forest Baptist Health, Department of Biochemistry, Winston-Salem, North Carolina 27157 US; 49 National Jewish Health, National Jewish Health, Denver, Colorado 80206 US; 50 Medical College of Wisconsin, Pediatrics, Milwaukee, Wisconsin 53226 US; 51 University of Texas Health at Houston, Pediatrics, Houston, Texas 77030 US; 52 University of California, San Francisco, San Francisco, California 94143 US; 53 Stanford University, Biomedical Data Science, Stanford, California 94305 US; 54 Brigham & Women’s Hospital, Brigham and Women’s Hospital, Boston, Massachusetts 2115 US; 55 University of Colorado at Denver, Denver, Colorado 80204 US; 56 Brigham & Women’s Hospital, Boston, Massachusetts 2115 US; 57 University of Montreal, US, Montreal, Canada; 58 University of Mississippi, Medicine, Jackson, Mississippi 39213 US; 59 Washington State University, Pullman, Washington 99164 US; 60 University of California, Los Angeles, Los Angeles, California 90095 US; 61 Brigham & Women’s Hospital, Medicine, Boston, Massachusetts 2115 US; 62 National Taiwan University, Taipei, 10617 TW China; 63 Brigham & Women’s Hospital, Division of Preventive Medicine, Boston, Massachusetts 2215 US; 64 University of Virginia, Charlottesville, Virginia 22903 US; 65 National Taiwan University, National Taiwan University Hospital,, Taipei, 10617 TW China; 66 National Health Research Institute Taiwan, Miaoli County, 350 TW China; 67 Cleveland Clinic, Immunity and Immunology, Cleveland, Ohio 44195 US; 68 University of Vermont, Burlington, Vermont 5405 US; 69 Boston University, Biostatistics, Boston, Massachusetts 2115 US; 70 University of Texas Rio Grande Valley School of Medicine, Brownsville, Texas 78520 US; 71 University of Michigan, Internal Medicine, Ann Arbor, Michigan 48109 US; 72 Vitalant Research Institute, San Francisco, California, 94118 US; 73 University of Illinois at Chicago, Chicago, Illinois 60607 US; 74 University of Chicago, Chicago, Illinois 60637 US; 75 Mayo Clinic, Health Quantitative Sciences Research, Rochester, Minnesota 55905 US; 76 Washington University in St Louis, Department of Medicine, Cardiovascular Division, St. Louis, Missouri 63110 US; 77 University of Texas Health at Houston, Human Genetics Center, Department of Epidemiology, Human Genetics, and Environmental Sciences, Houston, Texas 77030 US; 78 Vanderbilt University, Nashville, Tennessee 37235 US; 79 University of Cincinnati, Cincinnati, Ohio 45220 US; 80 University of North Carolina, Chapel Hill, North Carolina 27599 US; 81 University of Texas Rio Grande Valley School of Medicine, Edinburg, Texas 78539 US; 82 Washington University in St Louis, Genetics, St Louis, Missouri 63110 US; 83 Brown University, Providence, Rhode Island 2912 US; 84 Harvard University, Channing Division of Network Medicine, Cambridge, Massachusetts 2138 US; 85 Massachusetts General Hospital, Boston, Massachusetts 2114 US; 86 National Jewish Health, Center for Genes, Environment and Health, Denver, Colorado 80206 US; 87 University of North Carolina, Epidemiology, Chapel Hill, North Carolina 27599 US; 88 Washington University in St Louis, St Louis, Missouri 63130 US; 89 Fred Hutchinson Cancer Research Center, Seattle, Washington 98109 US; 90 Icahn School of Medicine at Mount Sinai, New York, New York 10029 US; 91 Boston Children’s Hospital, Harvard Medical School, Department of Psychiatry, Boston, Massachusetts 2115 US; 92 University of Texas Rio Grande Valley School of Medicine, San Antonio, Texas 78229 US; 93 University of Colorado Anschutz Medical Campus, Aurora, Colorado 80045 US; 94 Mass General Brigham, Obstetrics and Gynecology, Boston, Massachusetts 2115 US; 95 University of Calgary, Medicine, Calgary, CA Canada; 96 University of Maryland, Genetics, Philadelphia, Pennsylvania 19104 US; 97 Yale University, Department of Chronic Disease Epidemiology, New Haven, Connecticut 6520 US; 98 Tulane University, New Orleans, Louisiana 70118 US; 99 University of Washington, Epidemiology, Seattle, Washington 98195 US; 100 Wake Forest Baptist Health, Winston-Salem, North Carolina 27157 US; 101 Brigham & Women’s Hospital, Channing Division of Network Medicine, Boston, Massachusetts 2115 US; 102 University of Iowa, Iowa City, Iowa 52242 US; 103 National Health Research Institute Taiwan, Institute of Population Health Sciences, NHRI, Miaoli County, 350 TW China; 104 Tri-Service General Hospital National Defense Medical Center, Taipei, TW China; 105 Blood Works Northwest, Seattle, Washington 98104 US; 106 Taichung Veterans General Hospital Taiwan, Taichung City, 407 TW China; 107 Oklahoma State University Medical Center, Internal Medicine, DIvision of Endocrinology, Diabetes and Metabolism, Columbus, Ohio 43210 US; 108 Blood Works Northwest, Research Institute, Seattle, Washington 98104 US; 109 University of Michigan, Biostatistics, Ann Arbor, Michigan 48109 US; 110 Albert Einstein College of Medicine, New York, New York 10461 US; 111 University of California, San Francisco, San Francisco, California 94118 US; 112 Harvard University, Cambridge, Massachusetts 2138 US; 113 McGill University, MontrÃ©al, QC, H3A 0G4 Montreal, CA Canada; 114 University of Colorado at Denver, Epidemiology, Aurora, Colorado 80045 US; 115 Blood Works Northwest, Medicine, Seattle, Washington 98104 US; 116 Loyola University, Public Health Sciences, Maywood, Illinois 60153 US; 117 Harvard School of Public Health, Biostats, Boston, Massachusetts 2115 US; 118 University of Colorado at Denver, Medicine, Aurora, Colorado 80048 US; 119 Lundquist Institute, Torrance, California 90502 US; 120 Boston University, University of Massachusetts Chan Medical School, Worcester, Massachusetts 1655 US; 121 Harvard School of Public Health, Boston, Massachusetts 2115 US; 122 Brown University, Epidemiology and Medicine, Providence, Rhode Island 2912 US; 123 Stanford University, Cardiovascular Institute, Stanford, California 94305 US; 124 Icahn School of Medicine at Mount Sinai, The Charles Bronfman Institute for Personalized Medicine, New York, New York 10029 US; 125 Boston University, Boston, Massachusetts 2215 US; 126 Ohio State University, Division of Pulmonary, Critical Care and Sleep Medicine, Columbus, Ohio 43210 US; 127 Broad Institute, Harvard University, Massachusetts General Hospital, Boston, US; 128 George Washington University, cardiology, Washington, District of Columbia 20037 US; 129 Brown University, Epidemiology, Providence, Rhode Island 2912 US; 130 University of Washington, Department of Genome Sciences, Seattle, Washington 98195 US; 131 RTI International, Boston, US; 132 Massachusetts General Hospital, Medicine, Boston, Massachusetts 2114 US; 133 University of Arizona, Tucson, Arizona 85721 US; 134 Stanford University, Center For Sleep Sciences and Medicine, Palo Alto, California 94304 US; 135 National Institute of Child Health and Human Development, National Institutes of Health, Bethesda, Maryland 20892 US; 136 Oklahoma Medical Research Foundation, Genes and Human Disease, Oklahoma City, Oklahoma 73104 US; 137 Ministry of Health, Government of Samoa, Apia, WS Samoa; 138 Howard University, Washington, District of Columbia 20059 US; 139 University at Buffalo, Buffalo, New York 14260 US; 140 University of Pennsylvania, Division of Sleep Medicine/Department of Medicine, Philadelphia, Pennsylvania 19104-3403 US; 141 Stanford University, Stanford Cardiovascular Institute, Stanford, California 94305 US; 142 University of Minnesota, Minneapolis, Minnesota 55455 US; 143 RTI International, Biostatistics and Epidemiology Division, Research Triangle Park, North Carolina 27709-2194 US; 144 Boston University, Department of Biostatistics, Boston, Massachusetts 2118 US; 145 Fred Hutchinson Cancer Research Center, Fred Hutch and UW, Seattle, Washington 98109 US; 146 Johns Hopkins University, Cardiology/Medicine, Baltimore, Maryland 21218 US; 147 University of Colorado at Denver, Medicine, Denver, Colorado 80204 US; 148 University of Colorado at Denver, CCPM, Denver, Colorado 80045 US; 149 University of North Carolina, Genetics, Chapel Hill, North Carolina 27599 US; 150 Northwestern University, Chicago, Illinois 60208 US; 151 Fred Hutchinson Cancer Research Center, University of Washington, Seattle, Washington 98109 US; 152 Lutia I Puava Ae Mapu I Fagalele, Apia, WS Samoa; 153 University of Ottawa, Sleep Research Unit, University of Ottawa Institute for Mental Health Research, Ottawa, ON K1Z 7K4 CA Canada; 154 Vanderbilt University, Medicine, Pharmacology, Biomedicla Informatics, Nashville, Tennessee 37235 US; 155 Universidade de Sao Paulo, Faculdade de Medicina, Sao Paulo, 1310000 BR Brazil; 156 Columbia University, New York, New York 10027 US; 157 University of Maryland, Pathology, Seattle, Washington 98195 US; 158 Lundquist Institute, TGPS, Torrance, California 90502 US; 159 Harvard University, Division of Hematology/Oncology, Boston, Massachusetts 2115 US; 160 Harvard Medical School, Genetics, Boston, Massachusetts 2115 US; 161 Harvard Medical School, Boston, Massachusetts 2115 US; 162 Université Laval, Quebec City, G1V 0A6 CA Canada; 163 Emory University, Pediatrics, Atlanta, Georgia 30307 US; 164 Emory University, Human Genetics, Atlanta, Georgia 30322 US; 165 Vanderbilt University, Medicine/Cardiology, Nashville, Tennessee 37235 US; 166 UMass Memorial Medical Center, Worcester, Massachusetts, 1655 US; 167 University of Saskatchewan, Saskatoon, SK S7N 5C9 CA Canada; 168 Wake Forest Baptist Health, Biostatistical Sciences, Winston-Salem, North Carolina 27157 US; 169 University of Colorado at Denver, Genomic Cardiology, Aurora, Colorado 80045 US; 170 Brigham & Women’s Hospital, Channing Department of Medicine, Boston, Massachusetts 2115 US; 171 Stanford University, Genetics, Stanford, California 94305 US; 172 Fred Hutchinson Cancer Research Center, Cancer Prevention Division of Public Health Sciences, Seattle, Washington 98109 US; 173 University of Pennsylvania, Genetics, Philadelphia, Pennsylvania 19104 US; 174 University of Alabama, Biostatistics, Birmingham, Alabama 35487 US; 175 University of Southern California, USC Methylation Characterization Center, University of Southern California, California, 90033 US; 176 Brigham & Women’s Hospital, Mass General Brigham, Boston, Massachusetts 2115 US; 177 University of Pittsburgh, Department of Human Genetics, Pittsburgh, Pennsylvania 15260 US; 178 Brigham & Women’s Hospital, Channing Division of Network Medicine, Department of Medicine, Boston, Massachusetts 2115 US; 179 Indiana University, Epidemiology, Indianapolis, Indiana 46202 US; 180 Henry Ford Health System, Detroit, Michigan 48202 US; 181 University of Pittsburgh, Medicine, Pittsburgh, Pennsylvania 15260 US; 182 University of Michigan, Department of Epidemiology, Ann Arbor, Michigan 48109 US; 183 Case Western Reserve University, Department of Population and Quantitative Health Sciences, Cleveland, Ohio 44106 US; 184 University of California, San Francisco, US

**Keywords:** Genetic association study, Cardiovascular biology, Metabolomics, Genome-wide association studies

## Abstract

Integrating genetic information with metabolomics has provided new insights into genes affecting human metabolism. However, gene-metabolite integration has been primarily studied in individuals of European Ancestry, limiting the opportunity to leverage genomic diversity for discovery. In addition, these analyses have principally involved known metabolites, with the majority of the profiled peaks left unannotated. Here, we perform a whole genome association study of 2,291 metabolite peaks (known and unknown features) in 2,466 Black individuals from the Jackson Heart Study. We identify 519 locus-metabolite associations for 427 metabolite peaks and validate our findings in two multi-ethnic cohorts. A significant proportion of these associations are in ancestry specific alleles including findings in *APOE, TTR* and *CD36*. We leverage tandem mass spectrometry to annotate unknown metabolites, providing new insight into hereditary diseases including transthyretin amyloidosis and sickle cell disease. Our integrative omics approach leverages genomic diversity to provide novel insights into diverse cardiometabolic diseases.

## Abstract (web summary)

Most studies of the genetics of the metabolome have been done in individuals of European descent. Here, the authors integrate genomics and metabolomics in Black individuals, highlighting the value of whole genome sequencing in diverse populations and linking circulating metabolites to human disease.

## Introduction

Disturbed metabolism plays a central role across a spectrum of pathological processes from cancer to cardiometabolic disease^1,2^. Metabolomics aims to systematically measure small molecules and provides a snapshot of metabolic activity, capturing both genetic and environmental influences on disease pathogenesis^3^. The integration of genomics and metabolomics has been increasingly leveraged in efforts to identify bioactive metabolites linked to human disease, as large-scale genome-wide association studies (GWAS) have played a critical role in our understanding of loci that affect disease risk. Previous GWAS of the metabolome has identified associations between hundreds of genomic loci across a broad range of metabolite classes, including amino acids, nucleosides, and lipids among others^4–24^. While prior studies have ranged in sample sizes from several hundred to a few thousand individuals, a recent study performed a GWAS meta-analysis across several large cohorts for 171 metabolites (in up to 86,507 individuals) measured using various metabolomic profiling platforms, including liquid chromatography-mass spectrometry (LC-MS) and nuclear magnetic resonance (NMR) spectroscopy^23^. This cross-platform analysis highlighted the robustness of findings in metabolomics GWAS and the ability to detect clinically relevant associations, helping to illuminate biology in both common diseases such as diabetes and rare conditions such as macular telangiectasia type II.

Despite these efforts, the vast majority of metabolomics GWAS to date have been undertaken in cohorts of European ancestry, limiting the opportunity to leverage genomic diversity for biological discovery^25–27^. Individuals of African ancestry are more genetically diverse than those of European ancestry^28^, and carry ancestry-specific mutations which may illuminate biology and therapeutic strategies in cardiometabolic disease^29^. Further, most GWAS of the metabolome to date have used genotyping arrays with measurement of a limited set of tag single nucleotide polymorphisms (SNP) with the imputation of remaining variants, limiting the ability to accurately assess low-frequency protein-coding and non-coding variation. Finally, while tools for unbiased metabolomic profiling can now measure hundreds of known metabolites as well as thousands of unknown metabolite peaks^30,31^, the latter have eluded definitive compound identification, thus limiting biological insight into locus-metabolite associations. There are significant challenges in unknown metabolomic profiling, and attempts at further annotating these peaks in prior GWAS have been limited. First, unknown metabolite peaks must be separated from background noise and adduct ions of known and other unknown metabolites^32^. In addition, chemical identification of unique peaks requires downstream resource-intensive processes for structural elucidation, identification including the acquisition of product ion mass spectra (MS/MS) to generate metabolite fragmentation data as “chemical fingerprints” that can help improve compound identification^33^. We have previously demonstrated proof-of-principle suggesting the utility of integrating LC-MS of unknown peaks with genetic findings. For example, when peak levels map to solute carriers or enzymes with known functions, including substrates and products, this may help narrow the potential compound matches for chemical standard validation^34^. However, this has remained an arduous process, limiting its application in metabolomics GWAS of unknown peaks in large population-based studies.

To extend prior work, we performed a genome-wide association study integrating whole genome sequencing (WGS) of 2,291 metabolite peaks in 2466 participants from the Jackson Heart Study (JHS), a Black epidemiological cohort in Jackson, Mississippi, and validated findings in the Multi-Ethnic Study of Atherosclerosis (MESA; *n* = 995) and Health, Risk Factors, Exercise Training and Genetics Family Study (HERITAGE; *n* = 658). Beyond confirming prior known locus-metabolite associations in a Black cohort—an important next step to test the generalizability of prior work—we highlight many novel findings, including associations in ancestry-specific alleles for heritable conditions more commonly observed in Black individuals, including transthyretin amyloidosis and sickle cell disease. We acquired MS/MS on metabolite features and have integrated WGS findings and recently developed bioinformatic tools that leverage MS fragmentation data for more efficient annotation and identification of unknown metabolite peaks. We have developed and made available an extensive sample library of metabolite peaks, linking MS/MS spectra, genomic associations, and clinical traits that can be leveraged for annotation and identification of unknown metabolites implicated in diverse disease processes. Our integrative omics approach highlights the value of whole genome sequencing analysis of the metabolome in diverse populations for biological discovery and contributes to a roadmap for the identification of metabolites implicated in human disease.

## Results

**Fig. 1:**
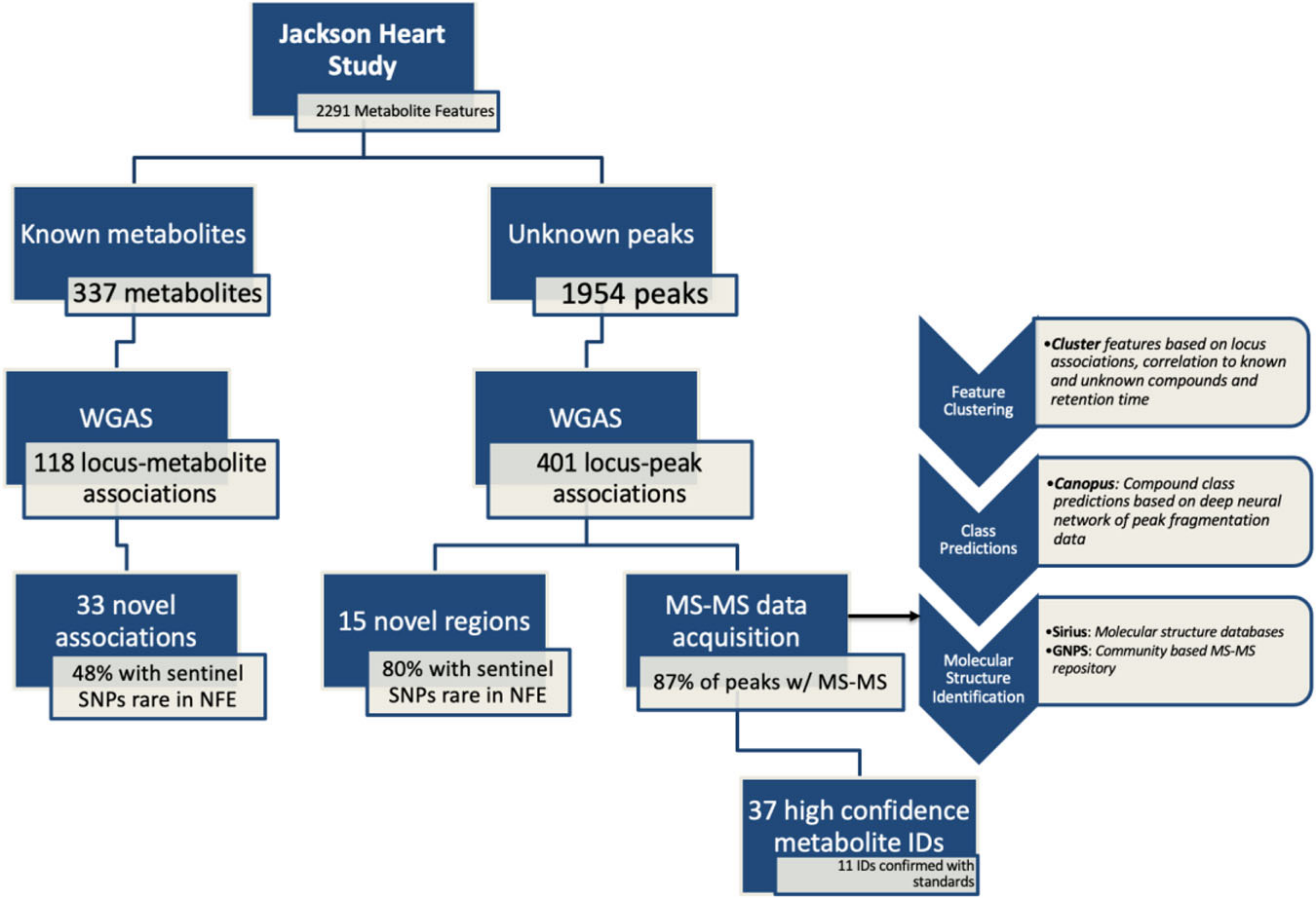
Whole Genome Association Study of known and unknown metabolites in the Jackson Heart Study. Flow diagram detailing whole genome association study of the metabolome, main results, and subsequent bioinformatic pipeline for unknown metabolite identification. Rare minor allele frequency is defined as <1% in NFE using gnomAD. Confirmation of metabolite identities was limited to commercially available metabolite standards. WGAS whole genome association study, MS mass spectrometry; NFE non-Finish Europeans, GNPS global natural product social networking.

**Fig. 2:**
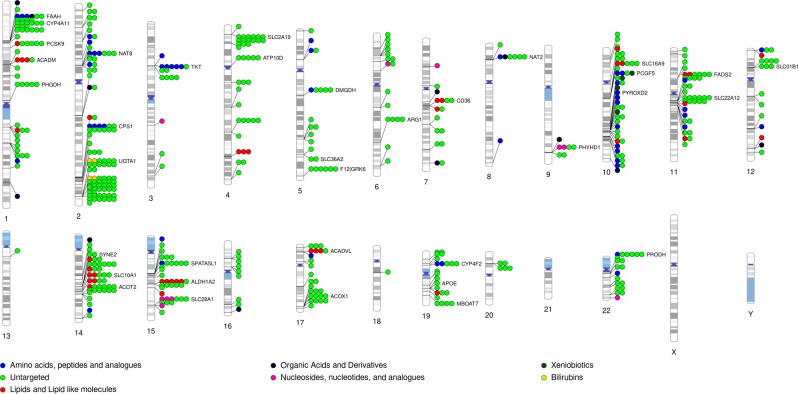
Phenogram of 519 locus-metabolite relationships in the Jackson Heart Study. 118 loci-metabolite associations are for known metabolites. 401 associations are for unknown metabolite features. The most common metabolite class includes amino acids, peptides, and analogs. Highlighted are sentinel genes with ≧4 locus-metabolite associations.

We performed a whole genome association study (WGAS) in 2466 Black participants from JHS on 30,672,656 variants limited to an allele count of at least five against 2291 metabolites (337 known metabolites and 1954 unknown metabolites peaks; Fig. 1). Clinical characteristics of the study population are detailed in Supplementary Table 1. At a Bonferroni threshold of significance of 8E-11 (based on 5E-8 /602 principal components, which explain 95% of the variance in metabolite peak levels), there were 519 locus-metabolite associations, representing 427 metabolite peaks and 226 sentinel SNPs (Fig. 2). Of these, 118 locus-metabolite associations were determined from known metabolite analysis, representing 91 distinct metabolites. Comparison to prior GWAS of plasma metabolites, using publicly available summary statistics through PhenoScanner V2^35^ and the GWAS Catalog^36^ as well as manual review of previously published metabolomics GWAS (Supplementary Methods), suggests 33 of these locus-known metabolite associations are novel. In addition, we identified 401 locus-metabolite associations from the unknown metabolite peak analysis, representing 336 metabolite features, highlighting a large amount of information in the yet-to-be-identified peaks.

**Fig. 3:**
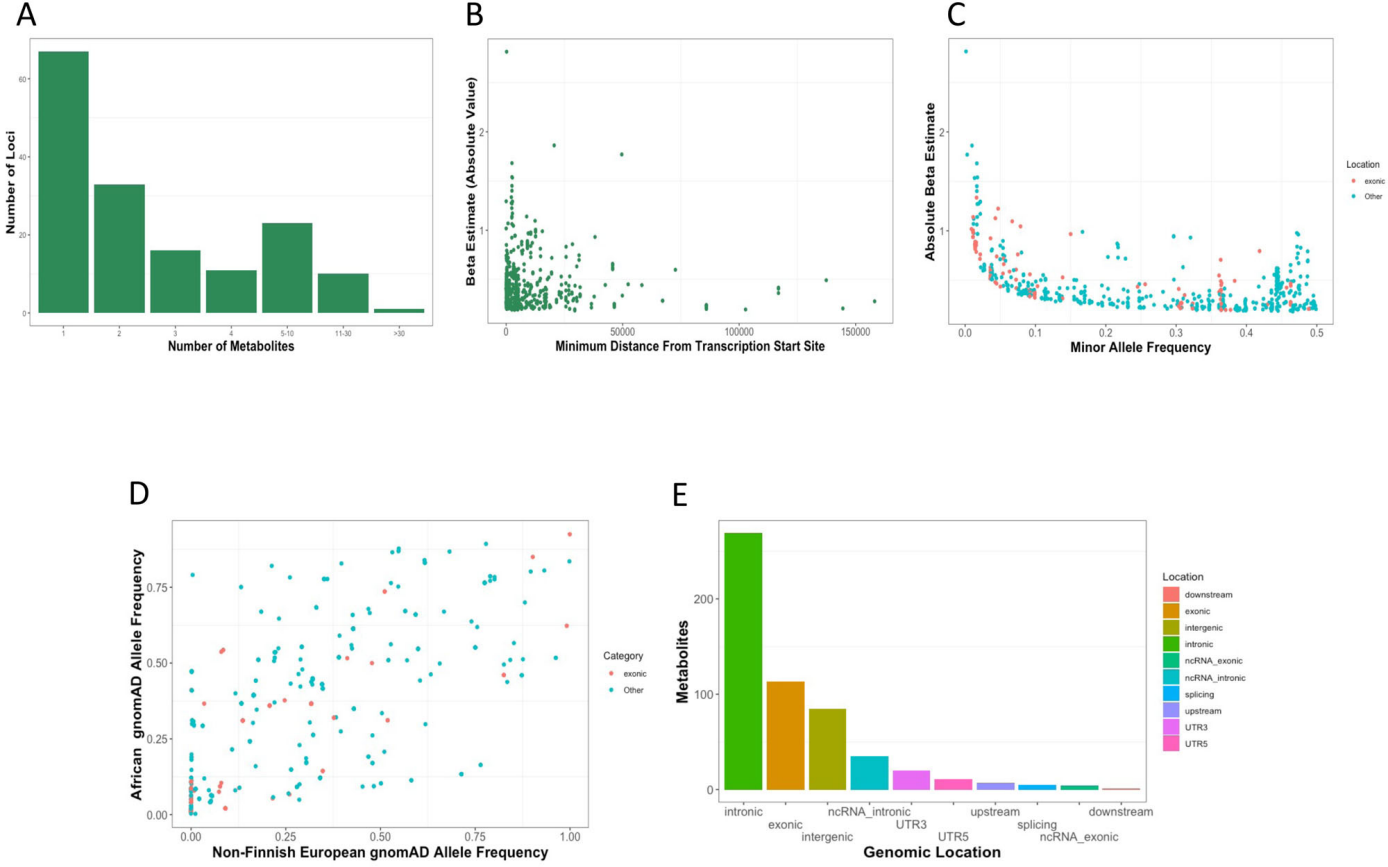
Genetic architecture of metabolite-WGAS associations. **A** Number of metabolites associated with each locus; **B** Absolute distance from mQTL position to transcription start site; **C** Minor allele frequency and effect size; **D** Frequency of mQTL sentinel allele in non-Finnish European Individuals vs African individuals; **E** Location of mQTL*.* WGAS whole genome association study, mQTL metabolite quantitative loci.

Of the 226 metabolite quantitative trait loci (mQTLs), there were 159 unique genes annotated as the lead candidate gene (closest protein-coding gene to the mQTL). Of the sentinel SNPs, 65% were expression quantitative loci (eQTL) for their corresponding gene as determined by PhenoScanner v2.0 (*p* value <2E-4; Supplementary Data 2). Of those that were not eQTLs for the candidate gene, the majority were rare in individuals of non-Finnish European Ancestry (MAF <1% in gnomAD), highlighting a key limitation of presently available genomic information when performing investigations in Black individuals. Among the sentinel SNPs, 22% were located in exons and an additional 19% were in enhancer or promoter regions (Fig. 3 and Supplementary Data 2).

Of the 519 locus-metabolite associations meeting the Bonferroni level of significance, 368 locus-metabolite peak relationships were available for validation in both MESA and HERITAGE, of which 91% were validated with a *p* value <0.05 and consistent direction of effect. An additional 100 locus-metabolite peak associations were available in *either* MESA or HERITAGE, of which 86% were validated. Overall, there was 90% validation of available locus-metabolite associations with *p* value <0.05; 68% validation (318 of 468 locus-metabolite associations available) using *Bonferroni* level of significance (0.05/468; *p* value <1E-4; Supplementary Data 2).

### Ancestry-specific variants and metabolite associations

WGS of diverse populations provides an opportunity to assess allelic architectures of populations of different ancestries and inform underlying biology. In particular, for our study, 17% of the 226 sentinel SNPs that were rare (MAF <1%) in Non-Finnish Europeans were common in individuals of African ancestry (MAF >5%; Supplementary Data 2). Overall, 29% of the sentinel SNPs were nearly monoallelic in individuals of NFE ancestry with a MAF <0.01% in gnomAD^37^. Here, we highlight several novel locus-metabolite relationships with SNPs that are rare in NFE (Table 1).

**Table 1:** Ancestry-specific mQTLs in the Jackson Heart Study

rsID	Position	Metabolite	Gene	Position	Allele	Beta	P Value	AFR AF
rs141239670	Chr1:171219209	Succinic acid	*SDHA*	Exon	T	1.01	4.4E-11	0.01
rs56072071	Chr2:215328065	AICA-riboside**	*ATIC*	Intron	A	0.38	2.9E-17	0.11
rs754490766	Chr3:51959148	*N*-acetylglutamic Acid	*PCBP4*	Intron	A	1.40	3.0E-36	0.015
rs754490766	Chr3:51959148	*N*-acetylserine	*PCBP4*	Intron	A	1.06	1.2E-21	0.015
rs73733867	Chr6:44207081	*N*4-acetylcytidine	*MYMX*	Intergenic	T	1.29	1.9E-37	0.02
rs3211938	Chr7:80671133	C38:6 PE plasmalogen	*CD36*	Exon	G	0.38	1.6E-15	0.09
rs3211938	Chr7:80671133	C38:7 PC plasmalogen	*CD36*	Exon	G	0.38	1.6E-15	0.09
rs115027210	Chr7:76062732	2-Hydroxyglutaric Acid	*MDH2*	Exon	C	0.53	4.6E-16	0.05
rs28832309	Chr7:80690622	C40:7 PE plasmalogen	*SEMAC3C*	Intergenic	C	0.35	2.3E-12	0.09
rs7079286	Chr10:106656814	*N*(6),*N*(6)-dimethyl-lysine	*F8MD8*	Exon	T	−0.48	1.2E-23	0.11
rs334	Chr11:5227002	LPC(OH-16:0)*	*HBB*	Exon	A	0.48	4.19E-11	0.04
rs624307	Chr11:65376604	3-Hydroxycarnitine	*SLC25A45*	Exon	T	−0.42	7.6E-14	0.08
rs12322356	Chr12:56378580	UDP-GlcNAc	*APOF*	Intergenic	C	0.29	6.0E-17	0.31
rs13333418	Chr16:30975943	Cholestanone**	*SETD1A*	Exon	C	−0.23	1.4E-13	0.30
rs28934585	Chr17:7220519	CAR 14:1	*ACADVL*	Exon	T	−0.37	3.0E-15	0.11
rs28934585	Chr17:7220519	CAR 14:2	*ACADVL*	Exon	T	−0.38	1.8E-14	0.11
rs28934585	Chr17:7220519	CAR 12:0	*ACADVL*	Exon	T	−0.35	2.5E-13	0.11
rs76992529	Chr18:31598655	All-trans retinol**	*TTR*	Exon	A	−0.76	4.6E-14	0.02
rs12721054	Chr19:44919330	DG (36:4)	*APOC1*	UTR	G	−0.31	1.3E-11	0.12

Novel, ancestry-specific mQTLs for known and unknown metabolites in the Jackson Heart Study (minor allele frequency for sentinel SNP less than 1% in non-Finish Europeans).

*AFR* African, *AF* allele frequency, *UTR* untranslated region.

*Unknown metabolite annotated using MS/MS data.

**Unknown metabolite annotated with MS/MS and confirmed with the chemical standard.

**Fig. 4:**
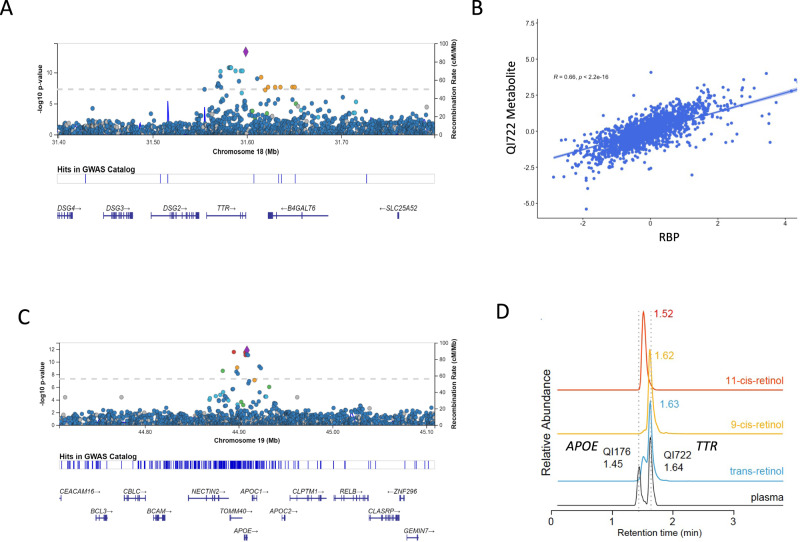
Ancestry-specific alleles reveal novel associations of TTR and APOE with retinol species. **A** Association of V122I in *TTR* with an unknown metabolite (QI722; *m/z 269.226*); **B** Correlation between QI722 and retinol-binding protein; **C** Association of rs769455 missense variant in *APOE* with unknown metabolite (QI176; m/z 269.226); **D** *TTR* associated unknown metabolite matching spectra with trans-retinol; *APOE* associated unknown metabolite with identical molecular mass but earlier retention time indicating it’s a *cis*-isomer of retinol. Additional isomers tested without compound match include 9 and 11-*cis* retinol.

As an example, The *TTR* variant (V122I) is present in 3–4% of Black individuals and has been implicated in the pathophysiology of heart failure in the elderly, often unrecognized. We found the V122I in *TTR* to be associated with an unknown metabolite (*m/z* 269.226) in JHS (ß = − 0.76, *p* value = 4.4E-14). The *TTR* tetramer complexes with retinol-binding protein (RBP4). This metabolite peak is correlated with RBP4 (*r*^2^ = 0.64) measured by aptamer-based proteomic profiling^38^ and V122I is significantly associated with RBP4 levels in JHS. Leveraging MS/MS data and its genetic association with *TTR* and correlation with RBP4, we predicted this compound to be all-*trans*-retinol (vitamin A), which we subsequently confirmed with an authentic standard (Supplementary Fig. 1). In addition, another variant that is nearly monoallelic in individuals of European ancestry, in *APOE* (rs769455), is associated with an unknown metabolite peak (*m/z* 269.226; ß = 0.71, *p* value = 1.5E-12) that has an identical molecular mass to all*-trans*-retinol (Fig. 4) and MS fragmentation analysis and MS comparison against chemical standards and all-*trans*-retinol and other retinol species predicts that it is a *cis*-isomer of retinol.

### Annotation for unknown metabolite peaks associated with genomic loci

**Fig. 5:**
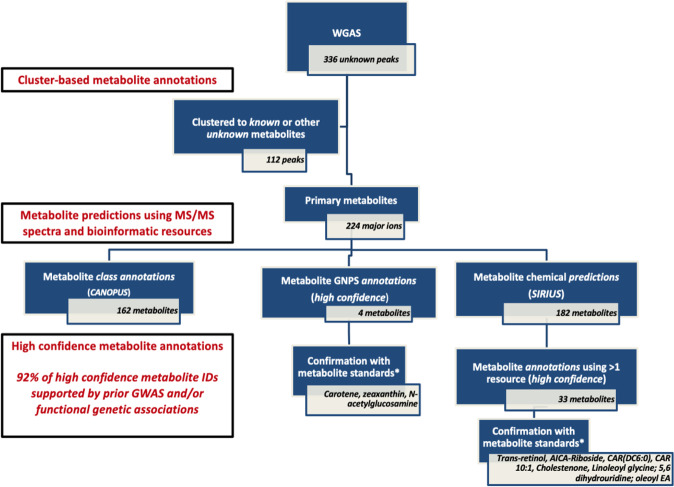
Unknown metabolite annotation pipeline using bioinformatic tools leveraging MS/MS spectra. Unknown metabolite identification with initial clustering of features to elucidate adducts and fragments of primary features or major ions. Subsequent implementation of tools leveraging MS/MS data, including SIRIUS, GNPS, and CANOPUS. Metabolite ID validation is limited to commercially available standards.

Of the 2291 metabolites measured, 1954 were unknown metabolite peaks, of which 336 were associated with genomic loci (*p* value <8E-11). Of the mQTLs associated with unknown features, 15 had no prior metabolite associations within 500 kb of the sentinel SNP; 12 of these SNPs were rare in individuals of NFE ancestry. (Supplementary Data 2). In the first step of assigning chemical identities to unknown metabolites, we clustered metabolite peaks measured in the positive mode (known and unknown metabolites) to identify primary metabolite features and their adducts (Supplementary Data 3). After applying our clustering algorithm, 49 of the 336 unknown metabolite peaks were part of clusters with known metabolites and were assigned the chemical identity of this primary metabolite. Of the 287 unknown metabolites not clustered with known compounds, 63 were adducts or fragments of other primary unknown metabolites, leaving 224 as major ions or primary unknown metabolites (Fig. 5).

Next, to aid in the identification of the unknown metabolites, we applied MS/MS profiling to metabolite peaks measured in positive mode, resulting in MS/MS data on 91% of unknown metabolite peaks designated as major ions or primary metabolites in our study. Using CANOPUS^39^, a bioinformatics tool which uses MS/MS data to annotate chemical compound class for metabolites, 72% of primary unknown metabolite features were assigned a metabolite compound class; lipids, amino acids, and fatty acids were the most common metabolite groups. SIRIUS^40^, a software tool that uses MS/MS data for metabolite structural elucidation, assigned chemical predictions in rank order for 182 primary unknown metabolites received (top three metabolite predictions for each feature in Supplementary Data 3). Leveraging these chemical predictions, we assigned high-confidence metabolite IDs to 33 metabolites that carried additional evidence for supporting the annotation using complementary tools, including Global Natural Product Social Molecular Networking (GNPS; *n* = 8)^41^, a database for MS/MS spectra in metabolomics studies, and the Human Metabolome Database (HMDB; *n* = 5)^32^, MS data for related compounds from our in-house metabolite library (*n* = 12) or validation with chemical standards (*n* = 8).

**Fig. 6:**
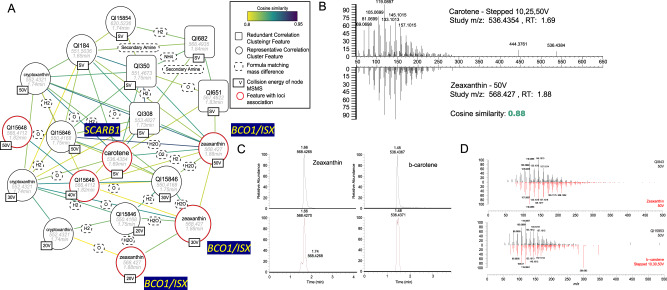
GNPS molecular network identifies carotenoid metabolites linked with genomic loci. **A** Molecular network of unknown features matching beta-carotene (*m/z* 536.4354; identified using MS/MS database) and carotene-related compounds using the Global Natural Products Social Molecular Networking. Nodes represent MS/MS spectra obtained at either discreet collision energies ranging from 10 to 50 V or stepped (SV) collision energies. The circular node shape illustrates whether features are representative ions (highest mean abundance) in clusters of co-eluting features with abundances correlating with Spearman coefficients >0.80. Conversely, square nodes correspond to features that based on correlation with co-eluting compounds, are potentially redundant fragments or adducts. Edges represent the cosine similarity among MS/MS spectra and formulas. Zeaxanthin is the predicted metabolite at *m/z* 568.427 based on *m/z* differences and association with *BCO1*, which catalyzes the conversion of carotenoids to retinal and *ISX*, which regulates the expression of *BCO1*. **B** Spectral comparison of plasma unknowns matching carotene and zeaxanthin MS/MS obtained using stepped and discrete collision energy, respectively, illustrating an edge cosine similarity score of 0.88. **C** Validation of compound identities for zeaxanthin and carotene confirming the retention time match of authentic standards with the unknown features in plasma (**D**) as well as their MS/MS spectrum match.

In addition to providing a database of MS/MS data for metabolomics studies, GNPS allows visualization of networks of structurally similar metabolites based on MS fragmentation data within studies to derive metabolite identities. As an example, chemical annotation using SIRIUS failed to generate a high-confidence metabolite prediction for an unknown metabolite with *m/z* 536.4354. However, using GNPS and comparing MS fragmentation data of this metabolite peak to community-based MS/MS spectra repositories, we were able to annotate this peak as carotene. Carotene is associated with rs2293440 (ß = 0.20, *p* value = 4.73E-11, an exonic variant in *Scavenger Receptor Class B Receptor 1 (SCARB1)*. SCARB1 plays a key role in lipoprotein metabolism through its action on reverse cholesterol transport and is associated with cholesterol levels in large population genomic studies^42^. Additionally, SCARB1 has been associated with the cellular uptake of carotenoids^43^, increasing our confidence in our metabolite annotation. Using GNPS, we mapped structurally similar unknown metabolites based on MS/MS spectra (Fig. 6A). A closely related metabolite peak (*m/z* 568.427*)* with a cosine similarity score for fragmentation spectra of 0.88 (Fig. 6B; high similarity score designated as >0.7) was noted to be associated with the *Beta-carotene 15,15-dioxegynase (BCO1)* and *Intestine Specific Homeobox (ISX)* loci. BCO1 converts carotenoids to retinal and ISX participates in carotenoid metabolism by regulating the expression of BCO1^44^. Anchoring our potential metabolite identification on these genomic associations, we searched through related carotenoid species based on mass differences between this peak and carotene and annotated this compound to be a carotenoid, zeaxanthin (Fig. 6D), which we subsequently validated with a chemical standard. In addition, the GNPS network identified another closely related compound in this network of unknown peaks, cryptoxanthin, a naturally occurring carotenoid compound.

Of the 37 high-confidence metabolite IDs from unique primary unknown metabolites (33 from SIRIUS predictions with supporting sources of information and four using GNPS; Supplementary Data 6), 92% (34/37) were evaluated as having genomic evidence in support of the chemical compound predictions based on the known metabolic pathways of the assigned lead candidate gene (Supplementary Data 2). In addition, CANOPUS annotated 29 of the 37 with metabolite class, of which 27 were in support of the metabolite annotation. We confirmed 11 unknown metabolite compound annotations with commercially available chemical standards; five of these are part of novel associations in a metabolomics GWAS: all-*trans*-retinol, zeaxanthin, 5,6 dihydrouridine, AICA-Riboside, and cholestanone (Supplementary Data 3 and Supplementary Fig. 1). MS/MS data on all metabolites associated with genomic loci in our study are made available (Supplementary Data 7).

## Discussion

In the present study, we used WGAS to identify genetic determinants of plasma metabolites in a Black population from JHS and applied novel chemical profiling and bioinformatic methods to annotate unknown metabolite peaks. In our cohort of Black individuals, the presence of ancestry-specific alleles that are nearly monoallelic in individuals of European ancestry increases the power to detect novel metabolomic associations with established cardiovascular risk loci—and represents an important first step in the broader discovery of ancestry-specific, pathogenically significant metabolic differences.

In this study, we show a novel association in a clinically relevant polymorphism in *TTR (Transthyretein)*, which is associated with increased risk of heart failure in Black individuals, and an unknown metabolite feature we identify as all-*trans-*retinol. The *TTR* V122I polymorphism described in 3–4% of Black individuals destabilizes the TTR-RBP4 tetramer, thereby displacing RBP4 and promoting amyloid fibril formation that precipitates heart failure and death^45^. The reduced circulating all-trans retinol observed in our study may similarly be related to increased clearance and a potential marker of TTR-RBP4 tetramer stability, as has been postulated in cases with ATTRv V122I amyloidosis^45^. The effect of reduced all-trans retinol on downstream active retinol metabolites, including retinoic acid, and its potential contribution toward pathologic cardiac hypertrophy needs further study.

The *Apolipoprotein E* locus is a complex genomic region encoding APOE and the isoforms produced from its polymorphic alleles. APOE is involved in critical metabolic pathways, including lipid transport and metabolism^46^, and is associated with chronic diseases, including the development of atherosclerosis and Alzheimer’s disease, presumably mediated by its role in the transport and clearance of cholesterol and amyloid peptides to the brain^47^. We show an association between a missense variant in *APOE* (rs769455), a nearly monoallelic polymorphism in individuals of European ancestry, with an unknown metabolite peak (*m/z* 269.226). This unknown metabolite has an identical molecular mass to all*-trans*-retinol and MS fragmentation analysis suggests that it is a potential isomer of retinol. While retinyl esters form with chylomicrons transported by APOE, the role of APOE transport on retinol species and potential downstream implications for retinoid transport and bioavailability has yet-to-be elucidated. Variants near retinoic acid transporters and receptors (downstream and active metabolites of retinol) have shown associations with risk for Alzheimer’s disease and defective transport of retinol and related species has been implicated in this disease^48,49^. Our findings showing associations between *APOE* and *cis*-retinol may have potential implications for the biological roles of APOE in disease pathways.

We show a novel association between an ancestry-specific variant in *CD36* (*Platelet Glycoprotein IV;* rs3211938) associated with specific plasmalogens, a subclass of phospholipids integral to cell membrane signaling and stability. CD36 is an integral transmembrane protein involved in the sequestration of malarial species, *plasmodium falciparum*, preventing splenic destruction of the organisms. As such, variants in *CD36* offer protection against malaria infection^50^. In addition, CD36 functions as a receptor for several important inflammatory mediators, fatty acids, and lipids among others, and is implicated in processes including regulation of blood pressure^51^, lipids^52^, and the development of atherosclerosis in model systems^53–55^. Novel associations of *CD36* with plasmalogen species, which are glycerophospholipids with important antioxidant properties and have been associated with the protection of endothelial cells in hypoxic conditions^56^, as well as with cell signaling and membrane stability^50^, highlight potential lipid mediators, and mechanisms for the role of CD36 in cardiometabolic disease development.

Sickle cell anemia is characterized by severe vascular abnormalities and leads to chronic cardiovascular diseases, including pulmonary hypertension, heart failure, and stroke^57^. In addition, individuals with the sickle cell trait are also at increased risk of developing chronic kidney disease^58^. Our findings show an association between the sickle mutation, *rs334*, and an unknown metabolite which we predict to be a lysophosphatidylcholine, a major component of red blood cell membranes. Red blood cell membrane structure is significantly altered in individuals with sickle cell disease, which affects cell shape, hemodynamics, and protein-membrane signaling interactions^59^. This metabolite association may be a marker of red blood cell membrane remodeling that occurs in sickle cell disease and may help elucidate mechanisms of red blood cell pathology and resulting downstream complications of ischemic and inflammatory tissue damage.

Unknown metabolomic profiling presents an opportunity for unbiased discovery in metabolomics GWAS. However, given the breadth and diversity of the metabolome, annotating metabolite peaks with subsequent validation of proposed metabolite identities is a lengthy and arduous process, traditionally requiring extensive manual curation of study features against reference databases. To facilitate a more efficient annotation pipeline for large-scale metabolomics GWAS, we performed additional tandem MS profiling to obtain MS fragmentation data for all our peaks and implemented recently developed bioinformatic methods that leverage MS/MS spectra in metabolomics studies to help annotate unknown compounds with chemical and/or class identities. Individual methods can help elucidate chemical identity by detailing compound sub-structure (SIRIUS), structural similarity to other metabolomic features (GNPS), and compound class (CANOPUS). In our study, no one method provided a complete annotation of study features, highlighting the challenges and complexity of working with unknown metabolomics. However, the use of complementary tools to elucidate metabolite identities enabled structural and/or class annotation for a majority of profiled peaks and represents the first systematic application of these bioinformatic tools to identify unknown peaks in a genomic association study.

We have previously demonstrated how genomic integration with MS fragmentation data on unknown metabolite peaks can help narrow the focus for these features, by mapping loci to predicted compounds based on shared metabolic pathways^34^. Here we have systematically integrated genetic associations with MS/MS spectra-based metabolite predictions from bioinformatic techniques. We find that a majority of our metabolite annotations, based on chemical identity, can be mapped to the metabolic/functional pathway of the associated lead candidate gene, providing an additional layer of support as we decipher metabolite identities associated with cardiometabolic diseases. As an example, an unknown metabolite feature with *m/z* 259.1036 is associated with triglycerides, DM, and CHD among other traits in JHS (Supplementary Data 4). This metabolite peak maps to the *ATIC* gene, which encodes *5-Aminoimidazole-4-Carboxamide Ribonucleotide Formyl transferase/IMP Cyclohydrolase*, a protein-coding gene involved in purine biosynthesis. One of the top computational predictions based on tandem MS for this metabolite was AICA-Riboside (Acedesine). While this compound has been described as an AMP-activated protein kinase agonist with investigational applications in treatments for diabetes and lymphoma^60–62^, there is evidence that endogenous levels of the compound are physiologically important. ATIC deficiency, a recessive genetic disease, results in impaired purine synthesis and increased urinary AICA-Riboside and is marked by severe neurodevelopmental delays, growth impairment, and dysmorphic features^63,64^. The association of AICA-Riboside, with its canonical enzymatic pathway in WGAS, narrowed our metabolite search to this single bioinformatic prediction, which we then confirmed with a commercial standard (Supplementary Fig. 1). Thus, the integration of genetics and MS/MS spectra can sometimes enable an efficient pipeline for identification of metabolites associated with cardiometabolic disease.

Our study represents one of the few analyses of genome-metabolome integration in a Black population. As such, validation of locus-metabolite associations presents a significant challenge, especially for associations in ancestry-specific alleles, given the scarcity of both known and unknown metabolomics profiling in Black populations. In addition, metabolomics GWAS have traditionally implemented genotype imputation of SNP array using reference panels. However, in an admixed population such as JHS, limited representative reference panels necessitate the use of more accurate imputation panels or whole genome sequencing. Further efforts to apply metabolomic profiling in Black populations and integrate with WGS will be essential to replicate key locus-metabolomic findings, though many have strong biologic plausibility. To assess the novelty of our findings, we used the most up-to-date genomic databases assessing genotype-phenotype associations, as well as a manual review of prior published metabolomics GWAS. However, there is the potential that we may have missed some previously published locus-metabolite associations. In addition, though we have made significant progress in compound identification for unknown metabolite features, a significant number still lack validation with chemical standards, and there may be some inaccuracies in chemical and/or compound class annotations, though we believe the integration of genetic findings and novel bioinformatic tools have helped minimize misclassification. While we will continue to systematically validate these compound IDs, we make available our sample library of MS/MS spectra with clinical and genomic associations, which can be leveraged across the omics community, providing a crowdsourcing opportunity for metabolite identification and serving as an ongoing resource for validation of metabolite peaks.

In summary, our integrative approach toward the identification of known and unknown metabolites involved in diverse disease processes using WGAS in a Black population highlights novel and clinically relevant locus-metabolite associations. In addition, genomic integration with advanced chemical phenotyping using tandem MS improves the ability to annotate unknown metabolite peaks, the “dark matter” of the metabolome. This sample library of MS/MS spectra of metabolite features linked to genomic loci and clinical traits will improve the identification of biologically relevant metabolites.

## Methods

### Cohorts

The study designs and methods for JHS, MESA, and HERITAGE have been described in refs. 65–67. JHS is a prospective population-based observational study designed to investigate risk factors for cardiovascular disease (CVD) in Black individuals. In 2000–2004, 5306 Black individuals from the Jackson, Mississippi tri-county area (Hinds, Rankin, and Madison counties) were recruited for a baseline examination. Of the original cohort, 2466 individuals had whole genome sequencing and metabolomic profiling performed from baseline fasting samples and were included in the analyses. MESA included 6814 participants between the ages of 45–84 years recruited at six clinical centers across the US, who were identified as members of four racial/ethnic groups: White, Hispanic, Asian, or Black (28%). Included in the present study are 995 individuals across all four racial/ethnic groups with metabolomic profiling and WGS at baseline exam. HERITAGE enrolled a combination of self-identified white and Black family units, totaling 763 sedentary participants (38% Black) between the ages of 17–65 years, in a 20-week, graded endurance exercise training study across four clinical centers in the US and Canada in 1995. Included in the present study is a random subset of 658 individuals with baseline metabolomic profiling and genotyping.

### Study approval

The Institutional Review Boards of Beth Israel Deaconess Medical Center, University of Mississippi Medical Center, University of Washington (MESA), and HERITAGE clinical centers approved the human study protocols, and all participants provided written informed consent.

### LC-MS metabolite profiling

Metabolite profiling was performed using two LC-MS methods. Organic acids and other intermediary metabolites were separated using amide chromatography (Waters XBridge Amide column) and measured using targeted negative ion mode multiple reaction monitoring (MRM) MS with an LC-MS system comprised of an Agilent 1290 infinity LC coupled to an Agilent 6490 triple quadrupole mass spectrometer. MRM data were processed using Agilent Masshunter QQQ Quantitative analysis software^68^.

Separately, amino acids, acylcarnitines, and other polar metabolites (including both known and unknown metabolite features) were separated using hydrophilic interaction liquid chromatography (HILIC) using an Atlantis HILIC column (Waters; Mildford, MA) and measured using nontargeted, full scan, high-resolution MS in the positive ion mode over *m/z* 70–800 with an LC-MS system comprised of a Nexera X2 U-HPLC (Shimadzu Corp.; Marlborough, MA) coupled to a Q Exactive mass spectrometer (Thermo Fisher Scientific; Waltham, MA). Raw data were processed using TraceFinder 3.3 (Thermo Fisher Scientific; Waltham, MA) for supervised integration of a subset of identified metabolites and quality control. Progenesis QI (Nonlinear Dynamics; Newcastle upon Tyne, UK) was used for the detection and integration of both identified and unknown features. Each feature in the dataset was tracked by its measured mass to charge ratio and chromatographic retention time, which serves as a unique “tag” for each LC-MS peak. Known compounds were annotated using mixtures of authentic reference standards analyzed with each batch and reference data. These metabolites had previously been annotated in human plasma and confirmed via spiking experiments with standards and by matching retention times and MS data. Metabolites with a coefficient of variation^69^ greater than 30% and those missing in more than 30% of measured samples were removed from the analysis^70^.

Isotope-labeled internal standards were monitored in each sample to ensure proper MS sensitivity for quality control. Pooled plasma samples were interspersed at intervals of 20 participant samples in the HILIC method and intervals of 10 participant samples in the amide chromatography method to enable correction of drift in instrument sensitivity over time and to scale data between batches. We used a linear scaling approach to the nearest pooled plasma sample in the queue. An additional pooled plasma sample was interspersed at every 20 injections to determine the coefficient of variation for each metabolite and unknown over the run. Peaks were manually reviewed in a blinded fashion to assess quality.

### MS/MS data acquisition

We acquired product ion mass spectra (MS/MS) on unknown features to aid their identification. All MS/MS data were acquired using an LC-MS system comprised of a Nexera X2 U-HPLC (Shimadzu Corp.; Marlborough, MA) coupled to an ID-X orbitrap mass spectrometer (Thermo Fisher Scientific; Waltham, MA). LC conditions were identical to those used in the nontargeted HILIC method, and electrospray ionization MS settings were spray voltage 3.5 kV, sheath gas 40, sweep gas 2, capillary temperature 350 °C, heater temperature 300 °C, S-lens RF 40. MS/MS data were generated using a combination of data-dependent acquisition (DDA) and inclusion list-directed MS/MS acquisition. For DDA, we used the AcquireX pipeline provided with the Thermo ID-X instrument and five consecutive injections of the JHS pooled plasma sample used for QC. The AcquireX scan cycle included an MS survey scan (70–800 m/z) followed by five MS/MS scans with a stepped collision energy of 10, 25, and 50 eV. To obtain MS/MS data on features not captured by the unsupervised AcquireX approach, we also used a directed MS/MS data acquisition approach in which lists of specific ions and retention time windows (inclusion lists) were created as required to measure spectra for ions of interest. First, we split all features in the study into 24 individual mass inclusion lists, separated based on ranges of metabolite peak retention times obtained from the initial LC-MS experiment, to improve the sensitivity of MS/MS data acquisition. We then generated MS/MS spectra using higher-energy C-trap dissociation (HCD) with stepped collision energies (10, 25, 50 V). Second, we targeted unknown features with GWAS hits and generated MS/MS with an expanded set of collision energies ranging from 10 to 50 V in 10 V increments. In order to increase the likelihood of capturing low abundance features in the JHS pool pooled plasma, samples used for MS/MS acquisition were concentrated ten-fold. Metabolites were extracted from 100 µL of pooled plasma using 900 µL of 74.9:24.9:0.2 (v/v/v) acetonitrile/methanol/formic acid. The samples were centrifuged (10 min, 9000×*g*, 4 °C) and the supernatants were dried under a gentle stream of nitrogen gas TurboVap LV, Biotage). Dried extracts were resuspended in 100 µL of 10:67.4:22.4:0.18 (v/v/v/v) water/acetonitrile/methanol/formic acid containing stable isotope-labeled internal standards (valine-d8, Sigma-Aldrich; St. Louis, MO; and phenylalanine-d8, Cambridge Isotope Laboratories; Andover, MA) and 10 µL were injected per LC-MS/MS analysis.

### MS/MS data processing

Raw files were converted to *.mzML format files using MSConvert^71^ and both extracted ion chromatograms^37^ and matching MS/MS scans for each individual feature were generated using the R package MSnbase v. 3.12^72^, and in-house scripts for producing EIC and MS/MS spectra visualizations. Feature retention times and peak quality in the concentrated pools were confirmed by visually inspecting the chromatography peak shapes of each individual feature. After confirming the study retention times in the MS/MS acquisition, the extraction of MS/MS data was conducted by finding scans with precursors within ±0.2 a.m.u. of the known features and ±0.1 min from the apex of the peak detected in the MS/MS run. Matching MS/MS peaks within 5 ppm across MS/MS scans spanning the range were aggregated whenever more than one MS/MS scan was mapped to each individual feature. The resulting peak height for aggregated peaks was determined as the average of the aggregated peak intensities. Peaks inconsistently detected across MS/MS scans were removed from the final MS/MS inventory. Additionally, an electronic noise fragment detected in the MS/MS of low abundance peaks within 30 ppm of *m/z* of 173.46 was removed from parsed data. Parsed MS/MS was formatted as input for molecular structure predictions (*.ms) or MS/MS-based similarity networks (*.MGF). For MS/MS-based similarity predictions, spectra generated for individual features using more than one collision energy were kept as independent molecular features.

### Genotyping

Whole genome sequencing (WGS) in JHS and MESA has been described in ref. 73. Participant samples underwent >30× WGS through the Trans-Omics for Precision Medicine project at the Northwest Genome Center at the University of Washington and the Broad Institute and joint genotype calling with participants in Freeze 6; genotype calling was performed by the Informatics Resource Center at the University of Michigan. Genotyping in HERITAGE was performed on the Illumina Infinium Global Screening Array. Genotypes were called using Illumina’s GenCall based on the TOP/BOT strand method. Genotype imputation to the TOPMed Freeze5 reference panel was performed using the University of Michigan Imputation Server Minimac4. In addition, phasing was performed with Eagle v2.4. Sites with call rates <90%, mismatched alleles, or invalid alleles were excluded.

### Whole genome association study

Metabolite LC-MS peak areas were log-transformed and scaled to a mean of zero and standard deviation of 1 and subsequently residualized on age, sex, batch, and principal components (PCs) of ancestry 1–10 as determined by the GENetic EStimation and Inference in Structured samples (GENESIS)^74^, and subsequently inverse normalized. The association between these values and genetic variants was tested using linear mixed-effects models adjusted for age, sex, the genetic relationship matrix, and PCs 1–10 using the fastGWA model implemented in the GCTA software package^75^. Variants with a minor allele count less than 5 in a given cohort were excluded from analysis in that cohort. A Bonferroni adjusted significance threshold of 8E-11 (5 × 10^−8^/602 PC’s explaining 95% of the variance of metabolite levels) was used for discovery in JHS. To identify sentinel SNPs and metabolite quantitative loci (mQTL), we first defined a 1 Mb region around each SNP significantly associated with a given metabolite. Starting at the SNP with the lowest *p* value, overlapping mQTLs for a particular metabolite were merged. This process was repeated until no more overlapping regions existed for the given metabolite, and the lead variant was identified as the one with the lowest *p* value. Lead variants that were not in overlapping regions but in linkage disequilibrium (LD) with *r*^2^ ≥ 0.8 were again combined using SNPClip^76^, and this final merged region was designated as the mQTL, with the most significant SNP retained as the sentinel variant. Where association statistics were available in both MESA and HERITAGE, the two cohorts were meta-analyzed by the inverse-variance weighted method using the “metagen” package in R. Validation threshold was set at *p* < 0.05 with a consistent direction of effect.

### Variant annotations

Reference allele frequencies from gnomAD and variant functional annotations using GENCODE and *ClinVar* disease annotations were obtained from the Functional Annotation of Variants—Online Resource (available favor.genohub.org, download date August 1, 2020).

### Comparing to previous mQTLs

We used existing genomic databases and prior blood metabolomics GWAS to assess the novelty of our locus-metabolite associations. To determine whether mQTLs of known metabolites were novel, we first utilized the PhenoScanner package for R. A 1 MB region around each sentinel SNP associated with a metabolite was passed to the PhenoScanner function in R: build was set to “38”, *p* value to 5 × 10^−8^, catalog to “mQTL” (query date 12/1/2021). Novel locus-metabolite associations were cross-referenced against the GWAS Catalogue using the sentinel SNP and a 1 MB surrounding region. In addition, gene-metabolite associations were manually reviewed for novelty across 21 published GWAS of metabolomics (details of individual studies reviewed in Supplementary Methods)^3–23^.

### mQTL and phenotype associations

To determine overlap between clinical GWAS analyses and mQTLs in this analysis, we utilized the PhenoScanner package for R. All sentinel SNPs associated with metabolite peaks as identified above were passed to the PhenoScanner function in R with the following arguments: build was set to “38”, *p* value to “1 × 10^−5^”, catalog to “GWAS”, r2 was set to “0.8”, proxies set to “None” (query date 12/1/2021).

### Unknown metabolite peak annotation

#### Metabolite peak clustering

The electrospray ionization process used in LC-MS can generate more than one type of ion adduct of a molecule (e.g., [M + H]^+^, [M + Na]^+^, etc.), partially fragment molecules, and generate multimer ions. A single metabolite may therefore give rise to multiple unknown peaks. However, such redundant features share the same chromatographic retention time and have highly correlated signal intensities. To filter redundant features, all profiled metabolite peaks (HILIC platform) were grouped into clusters based on a retention time similarity of ±0.25 min and a signal intensity spearman correlation coefficient >0.80. The [M + H] + ion, if identified by mass differences among features in the cluster or the feature with the highest signal intensity, was identified as the primary feature.

#### Metabolite annotations leveraging MS/MS spectra and bioinformatic tools

Parsed MS/MS data (*.ms) were loaded into SIRIUS CSI-Finger ID version 4.7.2^40^. Molecular formula predictions generated with Orbitrap-specific settings (MS/MS isotope scorer: ignore, mass deviation: 5 ppm, Candidates: 10, Candidates per ion: 1, possible ionizations: [M + H] + , [M + K] + , [M + Na]+). Structure elucidations were done using PubChem and the adducts [M + H] + , [M + K] + , [M + Na]+). Predictions were exported and the top three structure elucidations were parsed for each feature. Parsed MS/MS data for each metabolite peak were annotated for predicted metabolite class using ClassyFire ontology through CANOPUS^39^. MS/MS-based networks were built using the Global Natural Products Social Molecular Networking (GNPS)^41^, and the resulting networks were visualized with Cytoscape v. 3.8.2^77^. To provide supporting information for our metabolite annotations, we searched the Human Metabolome database with *m/z* ± 5 ppm^32^.

#### Metabolite annotation scheme

Results from metabolite feature clustering, class, and chemical structure elucidations were integrated to annotate metabolite peaks. We subsequently assigned each annotation to a category based on levels of supporting evidence. In addition, we classified the categories of metabolite identification in accordance with the Metabolomics Standards Initiative (MSI) recommendations (Supplementary Table 2):^78^ Category 1: metabolite match to an authentic reference standard; Category 2: metabolite clusters with a known compound which has previously validated with standard (Category 1 and 2 corresponds to MSI Classification 1 as Identified Metabolite); Category 3: metabolite with MS/MS-based GNPS database match (MSI Classification 2: putatively annotated metabolite); Category 4: Metabolite with similar MS/MS spectra and retention time with the representative backbone of chemical standard for a compound in the metabolite family (manual curation) using in-house metabolite library (MSI classification 3: putatively annotated metabolite class). In addition, we add category 5: SIRIUS MS/MS-based chemical formula/compound predictions and Category 6: compound match using *m/z* search in HMDB, which are not included in the 2007 MSI classification scheme. Primary unknown metabolites with multiple sources of supporting evidence represented high-confidence metabolite annotations and were assigned specific metabolite IDs.

#### Genomic and metabolite pathway integration

Genetic associations with unknown metabolite peaks can be integrated with MS/MS and bioinformatic metabolite annotations in an effort to further illuminate metabolite identifications and/or offer supporting evidence for predictions based on the known pathways of the locus. Each lead candidate gene (nearest gene to sentinel SNP) was annotated for its associated metabolic pathways using the KEGG database^79^. Metabolite annotations were evaluated in the context of the metabolic pathways of the candidate gene or its prior GWAS associations to assess whether there was genomic evidence in support of the chemical compound identification.

### Reporting summary

Further information on research design is available in the Nature Research Reporting Summary linked to this article.

## Supplementary information

Supplementary Info

Description of Additional Supplementary Files

Supplementary Data 1-7

Reporting Summary

## Data Availability

The WGAS summary statistics data generated in this study have been deposited in the GWAS catalog under accession code GCST90104476. Individual WGS data for TOPMed and metabolomic data for JHS and MESA, can be obtained by application to dbGaP with accession numbers for JHS and MESA are phs000964/phs002256.v5.p1 and phs001416.v2.p1. In addition, MS/MS spectra and analyses via Global Natural Product Structural Molecular Networking (GNPS) Job ID: aa6d11c8be15436abcb7d3d44fee5836 can be accessed at.
