# Supplementary material for: Whole Genome Association Study of the Plasma Metabolome Identifies Metabolites Linked to Cardiometabolic Disease in Black Individuals
Source: Nat Commun. 2022 Aug 22;13:4923. doi: 10.1038/s41467-022-32275-3 (PMC9395431; doi:10.1038/s41467-022-32275-3)
Supplement: Supplementary file 2 — Description of Additional Supplementary Files [file 41467_2022_32275_MOESM2_ESM.pdf]

Description of Additional Supplementary Files

File Name: Supplementary Data 1
Description: Metabolite reporting standards including quality control metrics.

File Name: Supplementary Data 2
Description: Whole genome sequencing analysis of plasma metabolome (519 locus-metabolite associations).

File Name: Supplementary Data 3
Description: Unknown metabolite annotations using bioinformatic tools (SIRIUS, GNPS, HMDB, In-house library).

File Name: Supplementary Data 4
Description: Associations between Unknown Peaks and Clinical Phenotypes (Jackson Heart Study).
File Name: Supplementary Data 5
Description: Phenotypic associations with sentinel SNP's using Phenoscanner V2.

File Name: Supplementary Data 6
Description: MS/MS fragmentation data for high condifence metabolite predictions. DDA: data dependent acquisition; RT: retention time.

File Name: Supplementary Data 7
Description: Metabolite ID-GNPS Node Map.
