# Supplementary material for: Whole Genome Association Study of the Plasma Metabolome Identifies Metabolites Linked to Cardiometabolic Disease in Black Individuals
Source: Nat Commun. 2022 Aug 22;13:4923. doi: 10.1038/s41467-022-32275-3 (PMC9395431; doi:10.1038/s41467-022-32275-3)
Supplement: Supplementary file 1 — Supplementary Info [file 41467_2022_32275_MOESM1_ESM.pdf]

Supplementary Material

1. Supplementary Table 1: Clinical characteristics in Jackson Heart Study
2. Supplementary Table 2: MSI and study-based metabolite classification
3. Supplementary Methods
4. Supplementary Figure 1: Unknown metabolite identification using chemical standards

Clinical Trait	Full Cohort (N=5301)	WGAS (N=2,466)
**Age, mean (SD)**	55.4 (12.8)	56 (12.7)
**BMI, mean (SD)**	31.8 (7.2)	31.7 (7.1)
**eGFR, mean (SD)**	85.7 (18.5)	85.5 (18.3)
**TC, mean (SD)**	199 (40.1)	199.8 (41)
**HDL, mean (SD)**	51.8 (14.6)	51.8 (14.7)
**sbp, mean (SD)**	127 (18.4)	127 (18.1)
**Male, n (%)**	1934 (36.5)	938 (38)
**Current Smoker, n (%)**	693 (13.1)	293 (11.9)
**HTN, n (%)**	3252 (61.3)	1530 (62)
**Diabetes, n (%)**	1152 (21.7)	506 (20.5)

Supplementary Table 1: Clinical characteristics between the entire Jackson Heart Study, available and profiled samples. BMI: body mass index; eGFR: estimated glomerular filtration rate; TC: total cholesterol, HDL: high density lipid lipoprotein; sbp: systolic blood pressure; HTN: hypertension

MSI Descriptor	MSI Classification	Study Classification
Class 1: Identified Metabolite	Match to an authentic reference standard	Category 1: Match to an authentic reference standard
		Category 2: Cluster with known compound (previously confirmed with standard)
Class 2: Putatively Annotated Metabolite	Matching physicochemical properties/similar spectra to known compound	Category 3: MS/MS match using GNPS database
Class 3: Putatively characterized metabolite class	Matching physicochemical properties/similar spectra to known class of compounds	Category 4: Spectra match to representative compound from metabolite family from in house library
N/A	N/A	Category 5: SIRIUS metabolite prediction
N/A	N/A	Category 6: HMDB match

Supplementary Table 2: Metabolomics Standard Inititative and Study Metabolite Classification Scheme. MSI: Metabolite Standard Initiative; HMDB: Human Metabolome Database

Supplementary Methods

Clinical Assessments and statistical analysis

Participants in JHS underwent a comprehensive history and physical exam at baseline exam. Current smoking status was obtained by questionnaire. Body mass index (BMI) was calculated using body weight indexed to height (kg/ m^2). Blood pressure was calculated by averaging two resting measurements. Hypertension was defined as systolic blood pressure of >140 mmHg, diastolic blood pressure >90 mmHg or use of anti-hypertensive medication. Diabetes mellitus status was determined by fasting glucose of >126 mg/dl, hemoglobin A1c >6.5% or use of anti-diabetic medication. High-density lipoprotein (HDL-C), total cholesterol, and creatinine were assayed directly using standard techniques[23]. Glomerular filtration rate was estimated by the chronic kidney disease epidemiologic collaboration (CKD-EPI) equation. Prevalent coronary heart disease status was assessed by questionnaire for history of myocardial infarction (MI), or evidence of MI on electrocardiogram. Associations between baseline clinical traits and non-targeted metabolite peaks were assessed using linear regression models adjusted for age, sex and batch (p value for significance (0.05/336 non-targeted metabolite peaks = 1.5E-4).

Review of prior metabolomics GWAS

A manual review of the literature was performed for blood metabolomics GWAS excluding populations enriched for background traits. Lead candidate gene and metabolomic associations were cross-checked for novelty. The details of the reviewed studies are found below:

Supplementary Table 3: Literature review of GWAS of blood metabolomics

Metabolomics	Genotyping	Number of Metabolites	Cohort Size	Study
Known	SNP array	363	284	Geiger et al. Plos Genetics, 2008
Known	SNP array	33 lipids +43 ratios	4,400	Hicks et al. Plos Genetics, 2009
Known	imputation	163 metabolites	1,809	Illig et al. Nature Genetics, 2010
Known+ Unknown	imputation	>250 metabolites	2,820	Suhre et. al. Nature, 2011
Known	imputation	216	8,330	Kettunen et al. Nature Genetics, 2012
Known	imputation	153 lipids	4304	Demirkan et al. Plos Genetics, 2012
Known	imputation	216	8330	Tukainen et al.,Human Molecular Gnetics 2012
Known	imputation	130	6600	Inoiuye et al., Plos Genetics2012
Known + unknown	imputation	517	1768	Krumsiek et al., Plos Genetics 2012
Known + unknown	imputation	163 + unknown peaks	1757	Rafler et al. Genome Medicine, 2013
Known	imputation	217	2076	Rhee et al, Cell Metabolism 2013
Known	imputation	308	1260	Yu et al., Plos Genetics2014
Known	WGS	102 lipids	3424	Fefanova et al., Genetics, 2018
Known + unknown	imputation	529	7824	Shin et al., Nature Genetics, 2014
Known	Imputation	96	2107	Burkhardt et al., Plos Genetics, 2015
Known	Exome	217	2016	Rhee et al., Nature Communications, 2016
Known	Imputation	340 lipids	5662	Harshfield et al., BMC Genomics, 2021
Known + unknown	Exome	826	614	Yousri et al, Nature Communications, 2018
Known	Imputation	640	3926	Fefanova et al., AJHG, 2021
Known + unknown	Imputation	1251	451	Barr et al, nature 2021

Known	Imputation	194	86,507 (meta-analysis)	Lotta et al., Nature genetics, 2021

Supplemental Figure 1: Confirmation of metabolite identities with chemical standards. Using commercially available standards, metabolite IDs were confirmed for 11 metabolites including 5,6 dihydrouridine, zeaxanthin, CAR(10:1), N-acetyl-D-galactosamine; oleoyl EA, CAR (DC6:0), linoleoyl glycine, beta-carotene; trans-retinol (testing of multiple retinol isomers against both QI176 and QI722 (trans-retinol), cholestanone and AICA-Riboside

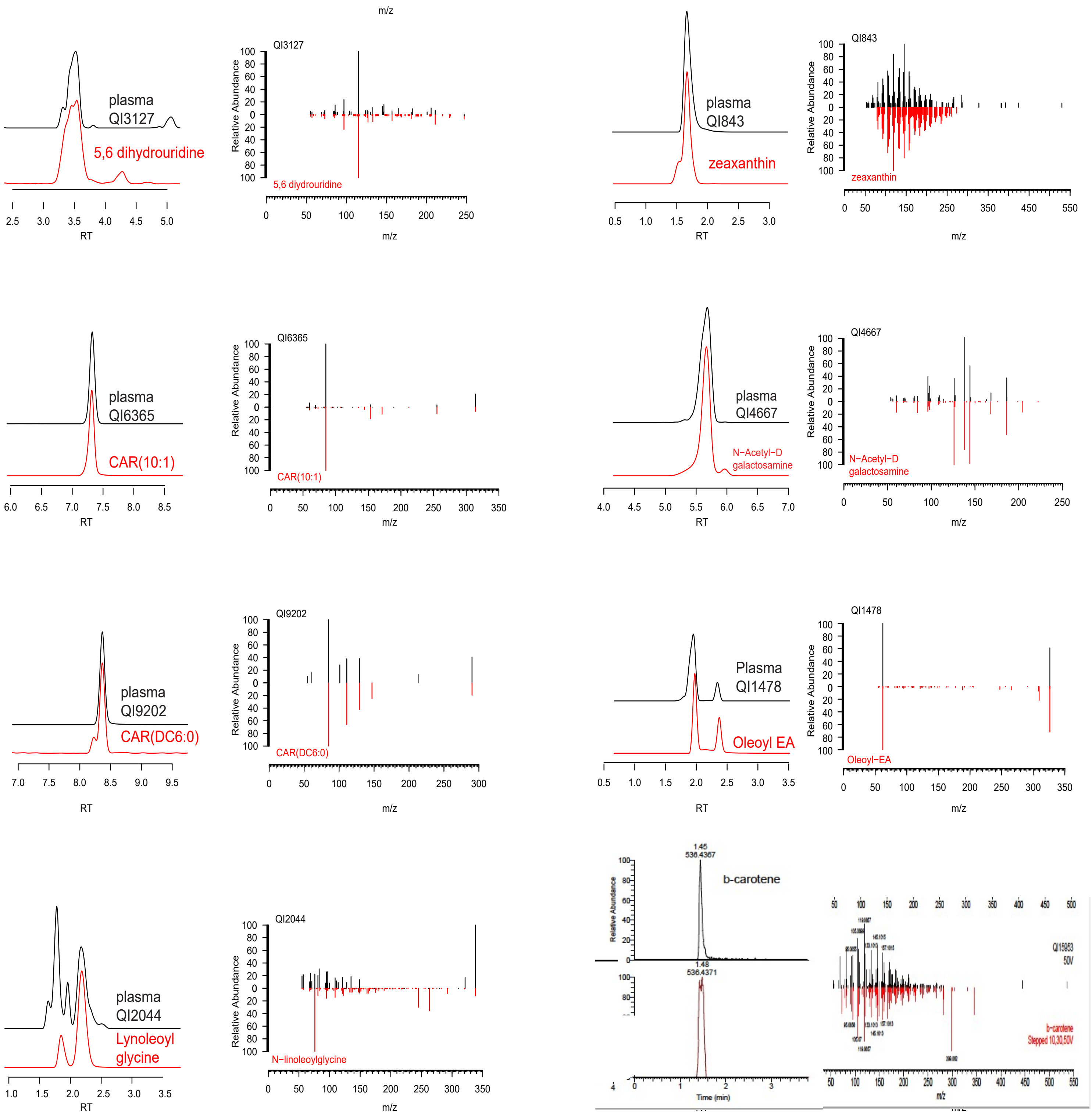

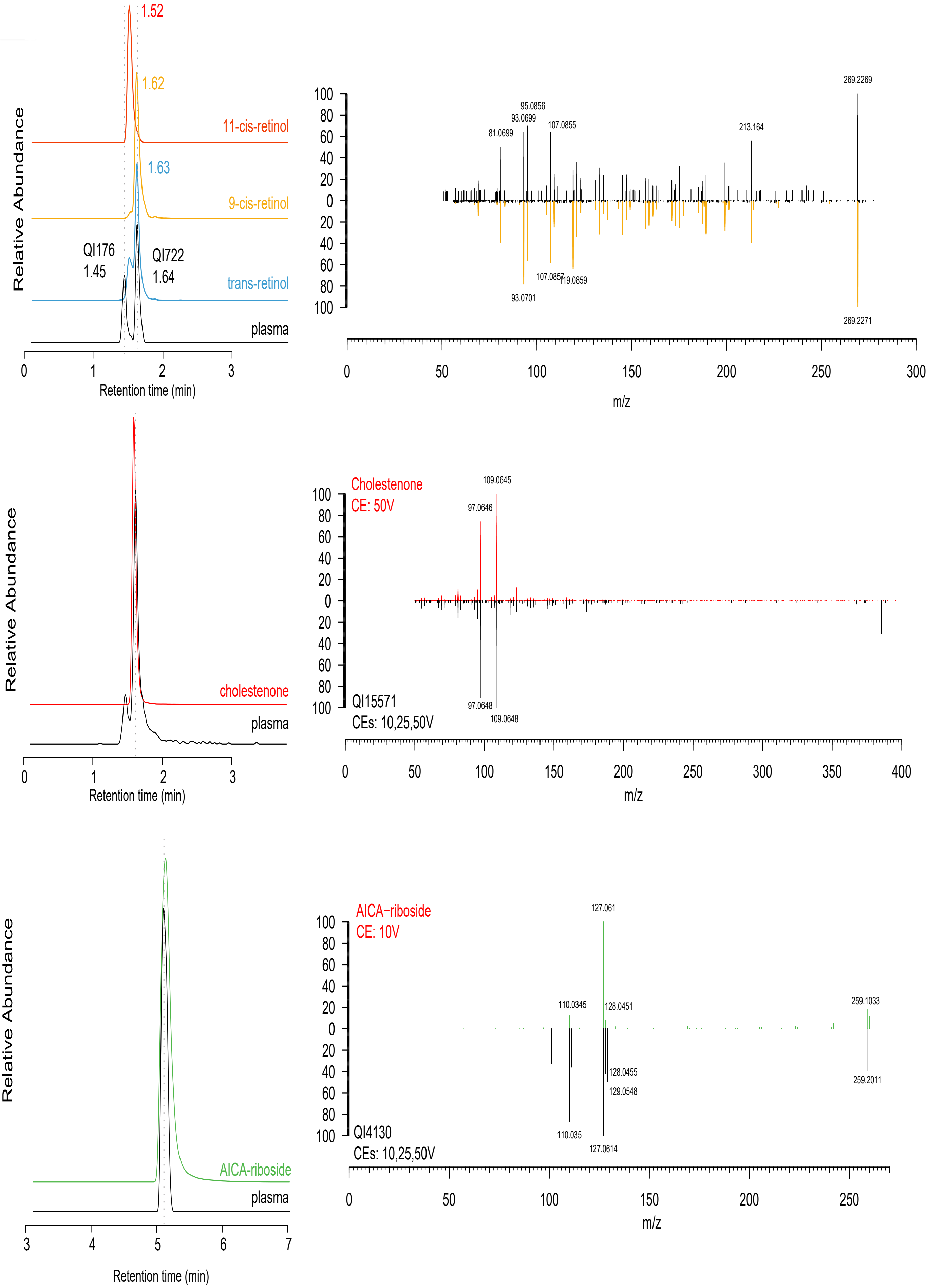
1.52
1.62
11-cis-retinol
1.63
9-cis-retinol
QI176
1.45
QI722
1.64
trans-retinol
plasma
Relative Abundance
Retention time (min)
269.2269
95.0856
93.0699
107.0855
81.0699
213.164
93.0701
107.0857
119.0859
269.2271
m/z
cholestenone
plasma
Cholestenone
CE: 50V
109.0645
97.0646
97.0648
109.0648
QI15571
CEs: 10,25,50V
AICA-riboside
plasma
AICA−riboside
CE: 10V
127.061
110.0345
128.0451
259.1033
128.0455
129.0548
259.2011
110.035
127.0614
QI4130
CEs: 10,25,50V
