# Supplementary material for: Whole Genome Association Study of the Plasma Metabolome Identifies Metabolites Linked to Cardiometabolic Disease in Black Individuals
Source: Nat Commun. 2022 Aug 22;13:4923. doi: 10.1038/s41467-022-32275-3 (PMC9395431; doi:10.1038/s41467-022-32275-3)
Supplement: Supplementary file 4 — Reporting Summary [file 41467_2022_32275_MOESM4_ESM.pdf]

nature portfolio

Corresponding author(s): Robert E. Gerszten

Last updated by author(s): 06/19/2022

# Reporting Summary

Nature Portfolio wishes to improve the reproducibility of the work that we publish. This form provides structure for consistency and transparency in reporting. For further information on Nature Portfolio policies, see our Editorial Policies and the Editorial Policy Checklist.

## Statistics

For all statistical analyses, confirm that the following items are present in the figure legend, table legend, main text, or Methods section.

| n/a | Confirmed | |
|---|---|---|
| ☐ | ☒ | The exact sample size (*n*) for each experimental group/condition, given as a discrete number and unit of measurement |
| ☐ | ☒ | A statement on whether measurements were taken from distinct samples or whether the same sample was measured repeatedly |
| ☐ | ☒ | The statistical test(s) used AND whether they are one- or two-sided<br>*Only common tests should be described solely by name; describe more complex techniques in the Methods section.* |
| ☐ | ☒ | A description of all covariates tested |
| ☐ | ☒ | A description of any assumptions or corrections, such as tests of normality and adjustment for multiple comparisons |
| ☐ | ☒ | A full description of the statistical parameters including central tendency (e.g. means) or other basic estimates (e.g. regression coefficient) AND variation (e.g. standard deviation) or associated estimates of uncertainty (e.g. confidence intervals) |
| ☐ | ☒ | For null hypothesis testing, the test statistic (e.g. *F*, *t*, *r*) with confidence intervals, effect sizes, degrees of freedom and *P* value noted<br>*Give P values as exact values whenever suitable.* |
| ☒ | ☐ | For Bayesian analysis, information on the choice of priors and Markov chain Monte Carlo settings |
| ☐ | ☒ | For hierarchical and complex designs, identification of the appropriate level for tests and full reporting of outcomes |
| ☐ | ☒ | Estimates of effect sizes (e.g. Cohen's *d*, Pearson's *r*), indicating how they were calculated |

*Our web collection on statistics for biologists contains articles on many of the points above.*

## Software and code

Policy information about availability of computer code

| | |
|---|---|
| Data collection | No software was used to collect data. |
| Data analysis | MSConvert was used to convert raw mass spectrometry data to .mzML files. SIRIUS 4 was used to elucidate chemical structures for unknown metabolites using MS/MS data. GNPS was used to perform an MS/MS database search and to assign molecular networks to unknown metabolite features. CANOPUS was used to assign chemical class to unknown metabolites using MS/MS data. |

For manuscripts utilizing custom algorithms or software that are central to the research but not yet described in published literature, software must be made available to editors and reviewers. We strongly encourage code deposition in a community repository (e.g. GitHub). See the Nature Portfolio guidelines for submitting code & software for further information.

## Data

Policy information about availability of data

All manuscripts must include a data availability statement. This statement should provide the following information, where applicable:

- Accession codes, unique identifiers, or web links for publicly available datasets
- A description of any restrictions on data availability
- For clinical datasets or third party data, please ensure that the statement adheres to our policy

WGAS summary statistics generated in this study are available through the GWAS Catalog (https://www.ebi.ac.uk/gwas/; Accession number GCST90104476). Individual WGS data for TOPMed and metabolomic data for JHS and MESA are available through dbGaP. Accession numbers for JHS and MESA are phs000964/phs002256.v5.p1 (https://www.ncbi.nlm.nih.gov/projects/gap/cgi-bin/study.cgi?study_id=phs000964.v5.p1) and phs001416.v2.p1 (https://www.ncbi.nlm.nih.gov/projects/gap/cgi-bin/study.cgi?study_id=phs001416.v2.p1), respectively. In addition, MS/MS spectra and analyses via Global Natural Product Structural Molecular

Networking (GNPS) Job ID: aa6d11c8be15436abcb7d3d44fee5836 can be accessed at: https://gnps.ucsd.edu/ProteoSAFe/status.jsp?task=aa6d11c8be15436abcb7d3d44fee5836.

# Field-specific reporting

Please select the one below that is the best fit for your research. If you are not sure, read the appropriate sections before making your selection.

☒ Life sciences ☐ Behavioural & social sciences ☐ Ecological, evolutionary & environmental sciences

For a reference copy of the document with all sections, see nature.com/documents/nr-reporting-summary-flat.pdf

# Life sciences study design

All studies must disclose on these points even when the disclosure is negative.

| | |
|---|---|
| Sample size | Sample size was determined by the availability of samples with both whole genome sequencing as well as metabolomics profiling. |
| Data exclusions | No data was excluded in the analysis. |
| Replication | Replication of findings in the Jackson Heart Study were carried out in the MESA and HERITAGE cohorts. |
| Randomization | Randomization is not relevant for this study given there was no control or experimental groups. |
| Blinding | Investigators involved in metabolomic profiling of study samples were blinded of sample identity and characteristics. |

# Reporting for specific materials, systems and methods

We require information from authors about some types of materials, experimental systems and methods used in many studies. Here, indicate whether each material, system or method listed is relevant to your study. If you are not sure if a list item applies to your research, read the appropriate section before selecting a response.

## Materials & experimental systems

| n/a | Involved in the study | |
|---|---|---|
| ☒ | ☐ | Antibodies |
| ☒ | ☐ | Eukaryotic cell lines |
| ☒ | ☐ | Palaeontology and archaeology |
| ☒ | ☐ | Animals and other organisms |
| ☐ | ☒ | Human research participants |
| ☒ | ☐ | Clinical data |
| ☒ | ☐ | Dual use research of concern |

## Methods

| n/a | Involved in the study | |
|---|---|---|
| ☒ | ☐ | ChIP-seq |
| ☐ | ☐ | Flow cytometry |
| ☒ | ☐ | MRI-based neuroimaging |

## Human research participants

Policy information about studies involving human research participants

| | |
|---|---|
| Population characteristics | Of the original cohort in Jackson Heart Study (5306), 2466 individuals had whole genome sequencing and metabolomic profiling performed from baseline samples and were included in the analyses. MESA included 6814 participants between the ages of 45-84 years recruited at six clinical centers across the US, who were identified as members of four racial/ethnic groups: White, Hispanic, Asian or Black. Included in the present study are 995 individuals across all four racial/ethnic groups with metabolomic profiling and WGS at baseline exam. HERITAGE enrolled a combination of self-identified white and Black family units, totaling 763 sedentary participants (62% white) between the ages of 17-65 years, in a 20-week, graded endurance exercise training study across four clinical centers in the US and Canada in 1995. Included in the present study is a random subset of 658 individuals with baseline metabolomic profiling and genotyping. |
| Recruitment | JHS is a prospective population based observational study designed to investigate risk factors for cardiovascular disease (CVD) in Black individuals. In 2000-2004, 5306 Black individuals from the Jackson, Mississippi tri-county area (Hinds, Rankin and Madison counties) were recruited for a baseline examination. MESA recruited at six clinical centers across the US, who were identified as members of four racial/ethnic groups: White, Hispanic, Asian or Black. HERITAGE enrolled a combination of self-identified white and Black family unitsacross four clinical centers in the US and Canada in 1995. |
| Ethics oversight | The National Health Lung, Blood Institute (NHLBI) approved this study. |

Note that full information on the approval of the study protocol must also be provided in the manuscript.

# Flow Cytometry

## Plots

Confirm that:

- [ ] The axis labels state the marker and fluorochrome used (e.g. CD4-FITC).
- [ ] The axis scales are clearly visible. Include numbers along axes only for bottom left plot of group (a 'group' is an analysis of identical markers).
- [ ] All plots are contour plots with outliers or pseudocolor plots.
- [ ] A numerical value for number of cells or percentage (with statistics) is provided.

## Methodology

| | |
|---|---|
| Sample preparation | *Describe the sample preparation, detailing the biological source of the cells and any tissue processing steps used.* |
| Instrument | *Identify the instrument used for data collection, specifying make and model number.* |
| Software | *Describe the software used to collect and analyze the flow cytometry data. For custom code that has been deposited into a community repository, provide accession details.* |
| Cell population abundance | *Describe the abundance of the relevant cell populations within post-sort fractions, providing details on the purity of the samples and how it was determined.* |
| Gating strategy | *Describe the gating strategy used for all relevant experiments, specifying the preliminary FSC/SSC gates of the starting cell population, indicating where boundaries between "positive" and "negative" staining cell populations are defined.* |

- [ ] Tick this box to confirm that a figure exemplifying the gating strategy is provided in the Supplementary Information.
